# An unconstrained four pool model analysis of proton relaxation and magnetization transfer in ex vivo white matter

**DOI:** 10.1038/s41598-025-87362-4

**Published:** 2025-02-05

**Authors:** Niklas Wallstein, André Pampel, Roland Müller, Carsten Jäger, Markus Morawski, Harald E. Möller

**Affiliations:** 1 NMR Methods and Development Group, Max Planck Institute for Human Cognitive and Brain Sciences, Stephanstr. 1A, 04103 Leipzig, Germany; 2 Department of Neurophysics, Max Planck Institute for Human Cognitive and Brain Sciences, Leipzig, Germany; 3 Medical Faculty, Paul Flechsig Institute – Centre of Neuropathology and Brain Research, Leipzig University, Leipzig, Germany; 4 Felix Bloch Institute for Solid State Physics, Leipzig University, Leipzig, Germany

**Keywords:** Four-pool model, Longitudinal relaxation, Magnetization transfer, Myelination, Transverse relaxation, White matter, Biophysics, Neuroscience

## Abstract

Understanding proton relaxation in the brain’s white matter remains an active field of magnetic resonance imaging research. Models of varying complexity have been proposed to link measurements to tissue composition/microstructure, in particular myelination. Although the presence of multiple aqueous and nonaqueous proton pools is well established experimentally, so-called “quantitative MRI” is usually based on simpler models due to the large number of model parameters. In this work, a comprehensive set of parameters characterizing a four-pool model is obtained. A piece of fixed porcine spinal-cord WM was investigated at 3 T and temperatures between 21 and 35 °C. Measurements included a wide range of preparations of the spin system in combination with long echo trains to achieve sensitivity to all model parameters. The results allow the extraction of all intrinsic relaxation and exchange rates as well as assigning them to specific dynamic processes involving tissue water. A critical assessment indicates that simpler models often lack specificity to myelin.

## Introduction

In magnetic resonance imaging (MRI), water proton relaxation is routinely used to generate contrast, for example, between cerebral white matter (WM) and gray matter. Relaxation parameters have also been increasingly used for quantitative analyses, particularly in terms of tissue microstructure or myelination patterns in the brain or spinal cord^1–7^. While it is often assumed that the longitudinal relaxation is characterized by a single rate ${R}_{1}=1/{T}_{1}$^8^ that correlates with myelination^9^, non-monoexponential recovery has been shown with sufficiently dense inversion-time sampling^3,10,11^. For transverse relaxation, due to its much faster “shutter speed”^12^, multiexponential behavior is well established and used to separate distinct water pools^13^. To reliably relate the results from relaxation measurements to tissue composition, a thorough understanding of the underlying processes that determine water-proton relaxation is, therefore, important^14^. This includes the consideration of magnetization-transfer (MT) processes between (mobile) aqueous and semisolid (motion-restricted) nonaqueous proton pools^2,5,11,15–18^.

Common simplifications in the analysis of transverse relaxation in the brain are to ignore nonaqueous pools and to assume fast chemical exchange of water between the intra- and extra-axonal spaces. This leads to two water pools, namely “myelin water” (MW) trapped between the myelin bilayers with a relaxation time ${T}_{2}$<40 ms and a single compartment of so-called “intra-/extracellular water” (IEW) with ${T}_{2}$ in the order of 40–90 ms^19–21^. The longitudinal relaxation dynamics, however, are not well captured if only aqueous protons are considered. Therefore, biophysical models of varying complexity have been utilized in studies of ${R}_{1}$^5,11,18^ and MT in WM^22–25^. The standard two-pool model (2PM)^22,26^ considers a single mobile pool ‘$A$’ of water protons and a semisolid pool ‘$B$’ of protons of macromolecules and membranes, which is typically referred to as “macromolecular pool”. Although it was successfully employed to describe experimental MT data in WM ex vivo^6^ and in vivo^24,25,27^, it does not account for separate aqueous reservoirs. In terms of a comprehensive expansion, it seems appropriate to assign separate semisolid pools to each of the water pools observed in transverse relaxation experiments, which leads to a four-pool model (4PM; Supplementary Figure S1)^2,5,18,23,28,29^.

Only a few studies^2,5,18,28^ have so far attempted to use this model. Stanisz et al.^28^ estimated 4PM parameters from MT-prepared acquisitions combined with a Carr–Purcell–Meiboom–Gill (CPMG) readout^30,31^ in fresh bovine optic nerve. More detailed insights into the relaxation and exchange processes were also obtained with an eigenvector interpretation^5,18^. By using dedicated schemes of “hard” and “soft” inversion radiofrequency (RF) pulses as well as Goldman-Shen (GS) filtering experiments^32^, Manning et al.^5^ generated six different initial conditions for the magnetization to elucidate how they affect longitudinal relaxation in heterogeneous systems. The results suggest that the 4PM is appropriate for interpreting ${T}_{1}$ and MT measurements. Nevertheless, all of the aforementioned studies also indicated that several parameters were poorly constrained by the available experimental data, highlighting the need for additional measurements and improved analysis concepts to fully characterize the model. This is not unexpected given the large number of unknowns and inherent parameter correlations, which include physically linked parameters, such as the fractional pool-sizes and exchange rates, as well as correlations arising within the fitting algorithm (e.g., between intrinsic relaxation rates).

**Fig. 1:**
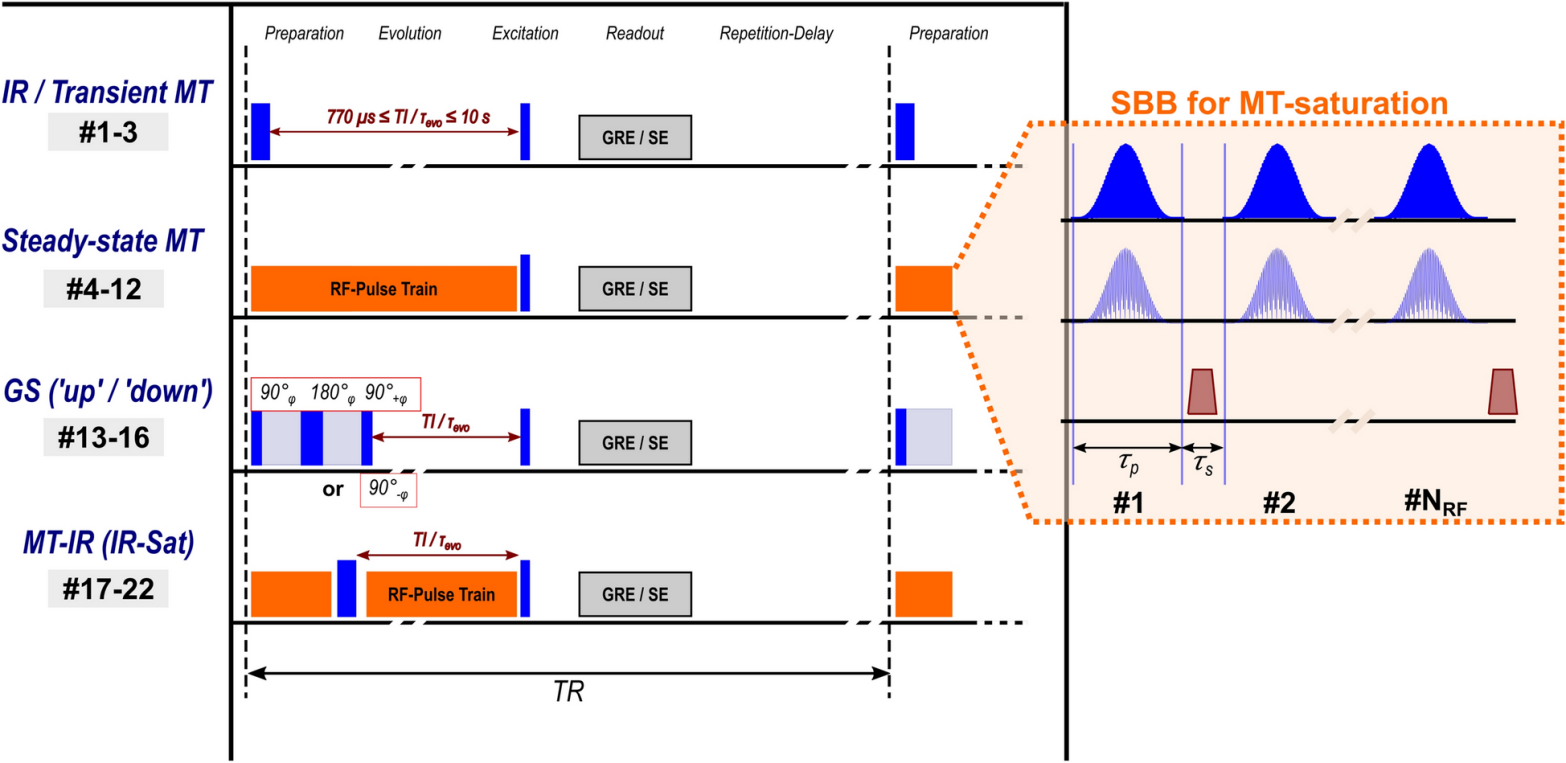
Diagrams of pulse sequences for relaxometry. They consist of a general scheme of sequence building blocks (SBBs). The individual types differ only in the *preparation* and *evolution* period. Acquisition parameters are summarized in Table 1. RF events are indicated as blue or orange rectangular boxes. For IR experiments (protocols 1 and 2), a rectangular or adiabatic pulse is utilized, while the magnetization is prepared by a composite MT pulse for transient MT experiments (protocol 3). Steady-state MT measurements (protocols 4–12) are performed using a saturation block (orange box), the details of which are shown in the enlarged insert on the right. It consists of ${{\varvec{N}}}_{\text{RF}}$ Gaussian-shaped, cosine-modulated pulses (second row of the insert) with pulse duration ${{\varvec{\tau}}}_{{\varvec{p}}}$ and inter-pulse delay ${{\varvec{\tau}}}_{{\varvec{s}}}$. Alternatively, off-resonance Gaussian pulses were also applied to acquire ${\varvec{z}}$-spectra (first row of the insert). The brown trapezoids indicate crusher gradients. The four types of the GS-filters (protocols 13–16) are shown as a simplified block with indication of the different pulse phases of the final 90° pulse to obtain GS ‘up’ or GS ‘down’. Finally, IR experiments were combined with the MT-saturation block (protocols 17–22). Here, the MT-saturation train (orange box) was applied before the inversion pulse and (whenever feasible) also during the evolution period.

The aim of this study was to extend previous experiments to obtain realistic estimates of *all* 4PM parameters without unverified assumptions. To achieve this, we used an unprecedentedly extensive set of measurements in fixed porcine spinal cord WM (Fig. 1 and Supplementary Table S1). These included MT-prepared acquisitions during a transient regime^33^, different inversion-recovery (IR) techniques^34^, with and without integrated pulsed MT saturation^35^, and GS filtering. All acquisitions were combined with an identical CPMG readout. We hypothesized that previously “inaccessible” 4PM parameters could be disentangled by an advanced analysis with simultaneous fitting of the entire experimental data. Measurements at different temperatures $T$ provided additional information about the dynamics of the tissue water. The consistency of the results was further checked by comparing forward simulations based on the fitted parameters with comprehensive MT measurements of so-called ‘z-spectra’^36^. Finally, transmission electron microscopy (TEM) was employed to assess the tissue microstructure of the sample.

## Results

### Tissue microstructure

**Fig. 2:**
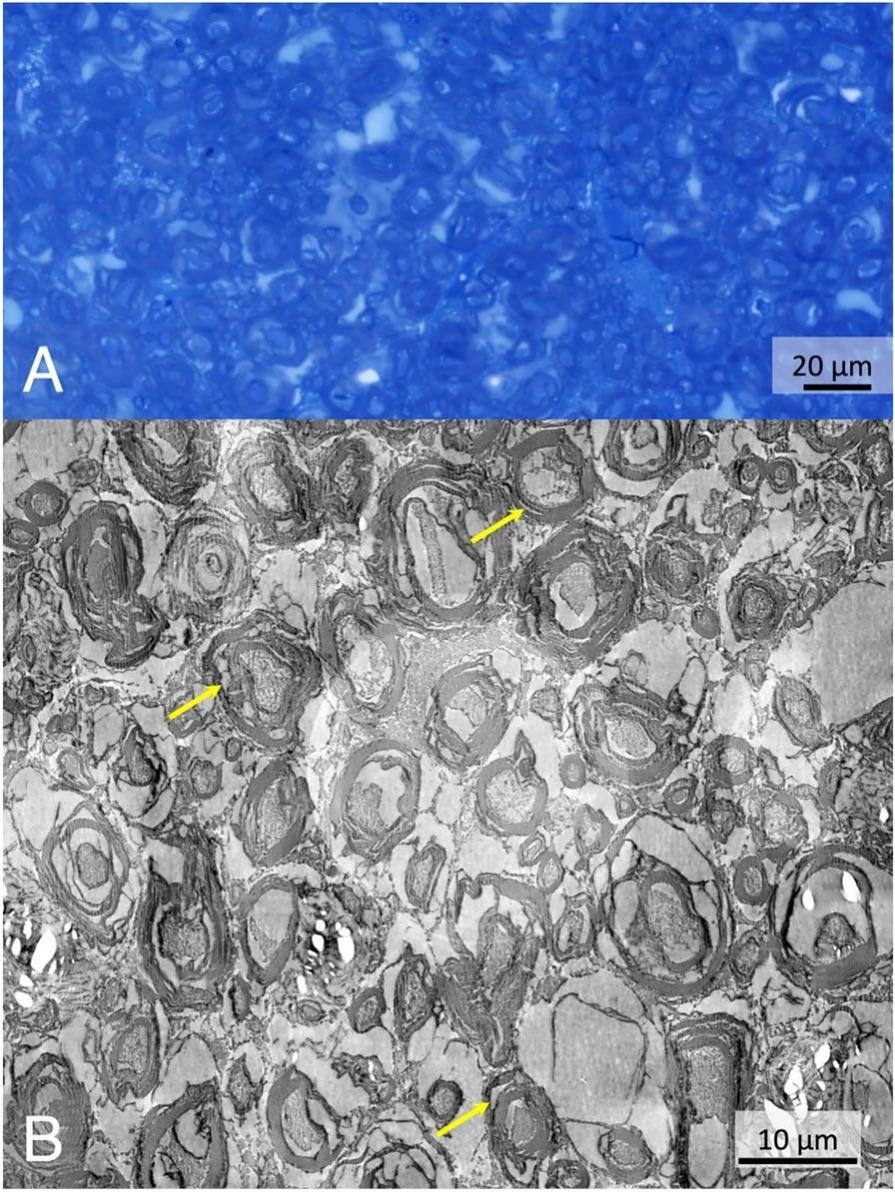
Light microscopy and transmission electron microscopy results. The Toluidine Blue-stained light microscopy image (**A**) of a semithin section shows highly ordered myelinated axons running perpendicular to the slice. The mosaic TEM image (80 kV, 3150 × magnification) of 12 × 12 tiles (**B**) reveals the presence of some vacuoles (indicated by yellow arrows), probably caused by inadequate PFA immersion fixation.

Analysis of diffusion-weighted imaging (DWI) data revealed a high fractional anisotropy (> 0.65) as expected for spinal cord WM. Results from fitting the DWI data to a scaled Bingham probability distribution (see Eq. 15) for extracting a fiber orientation distribution function (fODF) are shown in Supplementary Table S2. In summary, the fODF was adequately approximated by a single peak and a cylindrically symmetric distribution with so-called concentration parameters ${\kappa }_{1}$≈${\kappa }_{2}$≈5. A high degree of order of the fiber orientations running along the long axis of the cylindrical sample is also evident from examples of light and electron microscopy images presented in Fig. 2. Despite the relatively short *post-mortem* interval (compared to human tissue), delamination of the myelin sheaths and partial degradation of the axonal cytoplasm were observed for the majority of the axons as well as several vacuoles, probably caused by imperfect paraformaldehyde (PFA) immersion fixation. Similar observations have been reported in the literature for formalin-fixed human spinal-cord (immersion fixation) with *post-mortem* intervals > 18 h^37^ and occasionally even for rat spinal cord with minimal *post-mortem* intervals and perfusion fixation with 4% PFA/1% glutaraldehyde^38^. Based on the DWI results (i.e., high homogeneity with respect to fractional anisotropy values), a small region (7 voxels) almost in the center of the sample was selected for further analysis.

### 4PM analysis of relaxometry data

The 4PM (Supplementary Figure S1) contains two water pools (IEW and MW), each one connected to an individual semisolid pool, namely nonaqueous non-myelin (pool NM) and nonaqueous myelin (pool M)^5^. To account for a small amount of bulk water (BW) on the surface of the (wet) sample, another aqueous pool without exchange with any of the other reservoirs was further considered^5^. Each pool $l\in \left\{{\text{IEW}}, {\text{NM}}, {\text{MW}}, {\text{M}}, {\text{BW}}\right\}$ is characterized by a fractional size ${A}^{l}$ and relaxation rates ${R}_{\text{1,2}}^{l}=1/{T}_{\text{1,2}}^{l}$. Chemical exchange between the tissue water pools as well as MT between each water pool and its associated semisolid pool is modeled by fundamental rate constants ${k}_{{\text{IEW}}\leftrightarrow {\text{MW}}}$, ${k}_{{\text{IEW}}\leftrightarrow {\text{NM}}}$ and ${k}_{{\text{MW}}\leftrightarrow {\text{M}}}$. The fitting procedure considered the entire pulse sequence for each experiment using a matrix-algebra approach^24,39^ to solve the Bloch-McConnell Eqs. ^40,41^.

**Fig. 3:**
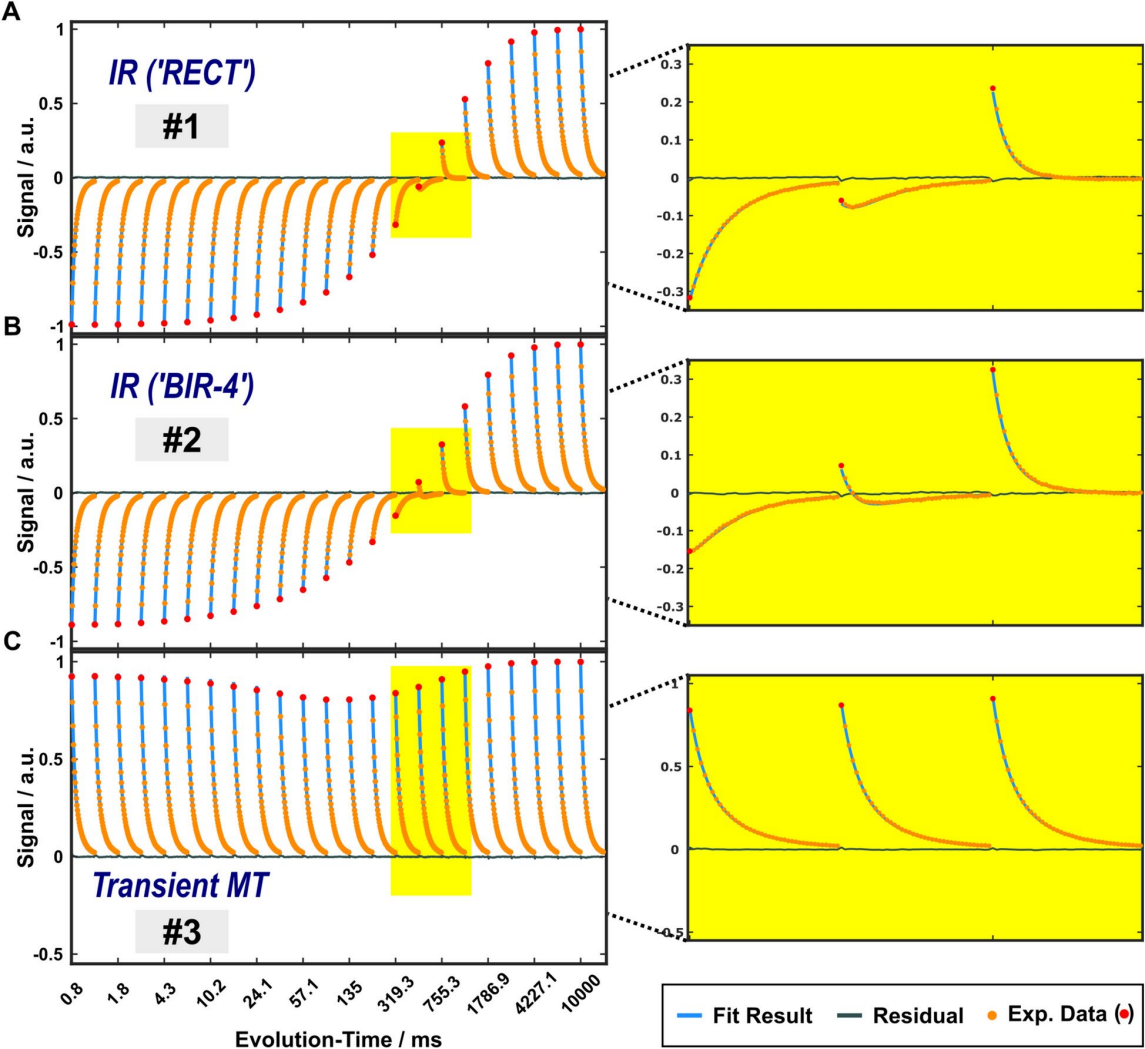
Examples of IR and transient MT experiments. The subset includes data recorded at 35 °C following (**A**) a 40 µs hard inversion pulse (‘RECT’, protocol 1) and (**B**) a 5 ms adiabatic inversion pulse (‘BIR-4’, protocol 2) as well as (**C**) transient MT preparation (protocol 3). The signals of the first echoes are shown as red circles and the remaining CPMG train as orange circles (only every second data point of the densely acquired echoes is plotted for better visibility). Results from fits to the 4PM (considering all acquisitions with protocols 1–22; Table 1) and residuals are indicated as solid blue and gray lines, respectively. Note that the evolution time is displayed on a logarithmic scale. The right column shows a zoomed region corresponding to the region indicated by yellow shading.

**Fig. 4:**
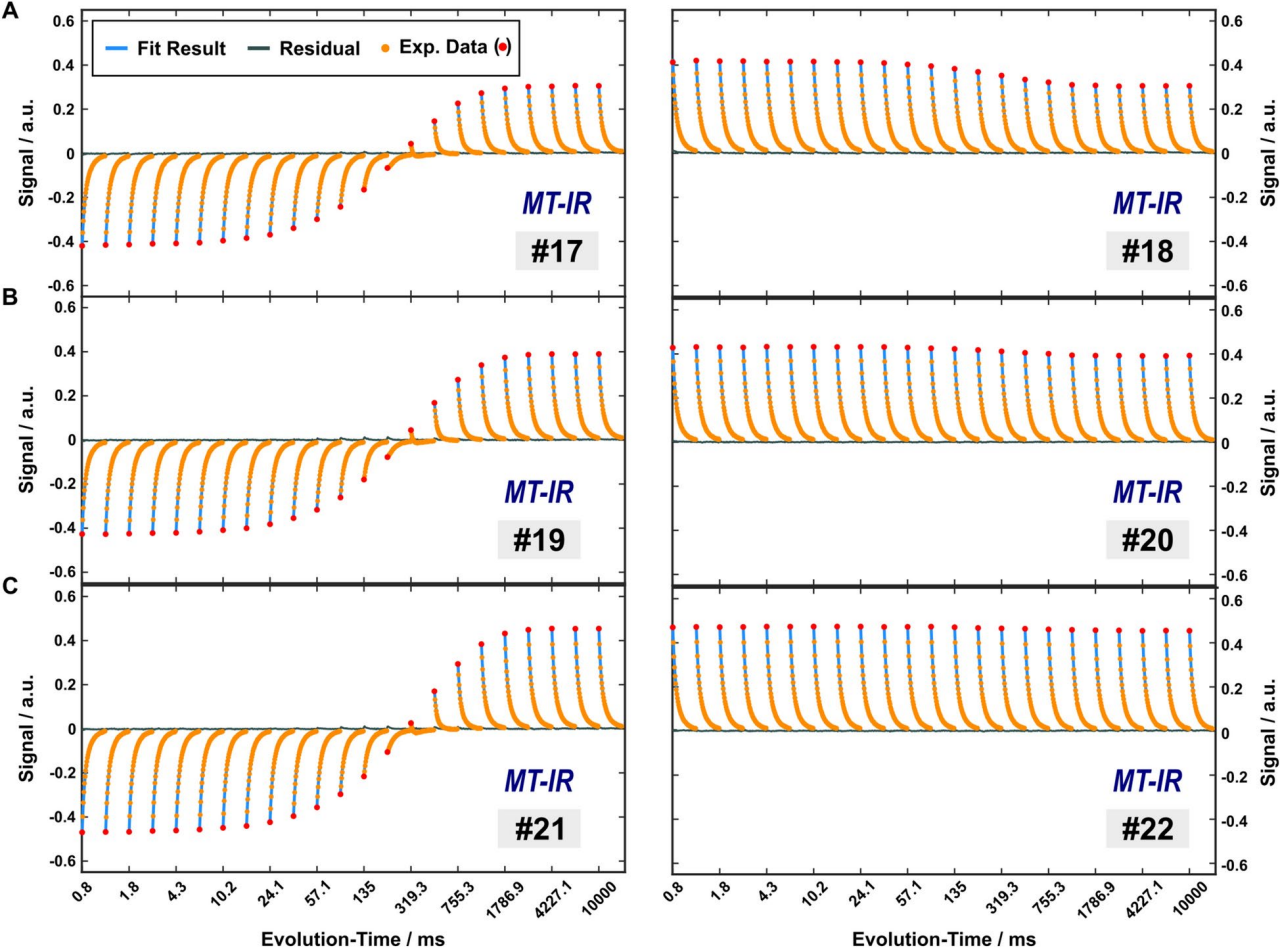
Examples of steady-state MT experiments. The subset includes data recorded at 35 °C after steady-state MT preparation with (left column; protocols 17, 19 and 21) and without (right column; protocols 18, 20 and 22) an additional 40 µs hard inversion pulse. The MT preparation was performed with increasing inter-pulse delays ${\tau }_{s}$ of (**A**) 0.25 ms, (**B**) 2.25 ms and (**C**) 4.25 ms, resulting in correspondingly reduced duty cycle and, therefore, reduced off-resonant saturation of the nonaqueous pools. Experimental data (red and orange circles), fits (blue solid lines) and residuals (gray solid lines) are displayed as in Fig. 4. Note that the residuals are almost invisible for most echoes and only slightly increased for TIs around the zero-crossing with the lowest SNR.

Plots from arbitrarily selected relaxometry experiments are shown in Fig. 3 (protocols 1–3), Fig. 4 (protocols 17–22) and Supplementary Figure S2. Fitted 4PM parameters are summarized in Table 1. Supplementary Table S4 shows additional results of effective flip angles ${\alpha }_{\text{eff}}^{\text{NM}}$ and ${\alpha }_{\text{eff}}^{\text{M}}$ for on-resonant RF pulses acting on the nonaqueous pools (obtained from fitting propagator elements ${\mathcal{P}}_{\text{5,5}}^{\left(i\right)}$ and ${\mathcal{P}}_{\text{9,9}}^{\left(i\right)}$ of the matrix-exponential solution to the Bloch-McConnell equations; see Eqs. 11 and 12). The assumption of temperature-independent fractional pool sizes improved the fit stability by reducing confounding correlations with the exchange rates. By contrast, separate fitting for each temperature led to variations in the pool-size estimates that exceeded the confidence intervals in Table 1 by a factor of ≈4. Correlations between fitted parameters are summarized in Supplementary Figure S3. The vast majority of correlations were weak (Pearson coefficients $\left|r\right|<\text{0.4}$), with some others moderate ($|r|<\text{0.6}$), suggesting well-determined estimates. The normalization ${\sum }_{l}{A}^{l}={1}$ produced intrinsic correlations between the fractional pool sizes ($\left|r\right|<\text{0.97}$), which should not be interpreted in terms of insufficient reliability. However, other anticorrelations appeared to indicate a lower accuracy in the simultaneous determination of selected parameters, as further analyses of residuals under variation of 4PM parameters suggest (Supplementary Figure S4). This includes primarily the exchange rates ${k}_{{\text{IEW}}\leftrightarrow {\text{NM}}}$ and ${k}_{{\text{MW}}\leftrightarrow {\text{M}}}$ as well as ${R}_{1}^{\text{NM}}$ and ${R}_{1}^{\text{M}}$ with $\left|r\right|>\text{0.8}$ and also (to a lesser extent) ${k}_{{\text{IEW}}\leftrightarrow {\text{MW}}}$ and ${R}_{1}^{\text{NM}}$ as well as ${R}_{1}^{\text{IEW}}$ and ${R}_{1}^{\text{MW}}$ with $\left|r\right|>\text{0.7}$.

**Table 1:** Summary of the fitted four-pool-model parameters from the combined analysis of the experiments performed at three temperatures.

Temperature-independent parameters			
Fractional pool sizes ${A}^{l}$		Corrected fractional pool sizes ${A}_{\text{c}}^{l}$ in WM	
${A}^{\text{IEW}}$	0.337 ± 0.011	${A}_{\text{c}}^{\text{IEW}}$	0.339 ± 0.011
${A}^{\text{MW}}$	0.431 ± 0.013	${A}_{\text{c}}^{\text{MW}}$	0.434 ± 0.013
${A}^{\text{NM}}$	0.044 ± 0.002	${A}_{\text{c}}^{\text{NM}}$	0.047 ± 0.001
${A}^{\text{M}}$	0.181 ± 0.001	${A}_{\text{c}}^{\text{M}}$	0.181 ± 0.003
${A}^{\text{BW}}$	0.007 ± 0.001*		–
${B}_{1}^{+}$ scaling factor			
${f}_{B1}$	0.985 ± 0.001		

Temperature-dependent parameters			
	21 °C	28 °C	35 °C
Exchange rates ${k}_{l\leftrightarrow m}$			
${k}_{{\text{IEW}}\leftrightarrow {\text{MW}}}$/s^–1^	1.10 ± 0.06	1.38 ± 0.07	1.86 ± 0.08
${k}_{{\text{IEW}}\leftrightarrow {\text{NM}}}$/s^–1^	42.26 ± 0.93	45.16 ± 1.05	47.99 ± 1.10
${k}_{{\text{MW}}\leftrightarrow {\text{M}}}$/s^–1^	36.49 ± 0.54	32.53 ± 0.50	33.99 ± 0.53
Longitudinal relaxation rates ${R}_{1}^{l}$			
${R}_{1}^{\text{IEW}}$/s^–1^	0.63 ± 0.01	0.57 ± 0.01	0.52 ± 0.01
${R}_{1}^{\text{MW}}$/s^–1^	1.40 ± 0.02	1.28 ± 0.02	1.26 ± 0.02
${R}_{1}^{\text{NM}}$/s^–1^	2.84 ± 0.12	2.68 ± 0.13	2.67 ± 0.14
${R}_{1}^{\text{M}}$/s^–1^	3.52 ± 0.08	3.78 ± 0.10	3.38 ± 0.10
${R}_{1}^{\text{BW}}$/s^–1^	*(0.44* ± *0.02)*^†^	*(0.44* ± *0.02)*^†^	*(0.44* ± *0.02)*^†^
Transverse relaxation rates ${R}_{2}^{l}$ (aqueous pools)			
${R}_{2}^{\text{IEW}}$/s^–1^	7.08 ± 0.03	7.59 ± 0.03	8.19 ± 0.03
${R}_{2}^{\text{MW}}$/s^–1^	24.00 ± 0.09	25.00 ± 0.09	26.85 ± 0.10
${R}_{2}^{\text{BW}}$/s^–1^	*(0.50* ± *0.10)*^†^	1.16 ± 0.11	*(2.00* ± *0.11)*^†^
Transverse relaxation times ${T}_{2}^{l}$ (nonaqueous pools)			
${T}_{2}^{\text{NM}}$/µs	13.04 ± 0.10	12.76 ± 0.10	12.68 ± 0.11
${T}_{2}^{\text{M}}$/µs	13.31 ± 0.07	13.74 ± 0.08	14.30 ± 0.08

The total number of fitted parameters was 44. Since only $\Delta \nu$ =  ± 15 kHz was utilized in the acquisition of relaxometry data (Supplementary Table S1; protocols 17–22), the fits did not require the assumption of a specific absorption lineshape for the nonaqueous pools. For easier comparisons to literature values, ${T}_{2}^{\text{NM}}$ and ${T}_{2}^{\text{M}}$ correspond to a super-Lorentzian, Eq. 11, which is typically assumed for WM. Uncertainties represent the 95% confidence interval.

*The confidence interval was not obtained directly from the fit but estimated by error propagation.

^†^The estimated relaxation time of the BW pool assumed a boundary value.

Significance value italics.

The estimated ${A}^{\text{BW}}$<1% indicated that water on the sample surface had negligible impact. As the BW pool is not part of the WM, the pool-size fractions were corrected according to ${A}_{c}^{l}={A}^{l}/{\sum }_{l\ne {\text{BW}}}{A}^{l}$ to represent only the tissue. Remarkably, the IEW and MW pools were of similar size, while the corresponding NM and M pools differed by a factor of ≈2.5.

Focusing on the results obtained at 35 °C, water exchange between the IEW and MW pools (${k}_{{\text{IEW}}\leftrightarrow {\text{MW}}}$≈1.9 s^–1^) was the slowest exchange process, whereas MT between the aqueous and their associated nonaqueous pools was an order of magnitude faster ($k_{{{\text{MW}} \leftrightarrow {\text{M}}}}$≈34 s^–1^ and $k_{{{\text{IEW}} \leftrightarrow {\text{NM}}}}$≈48 s^–1^). Note that the difference between $k_{{{\text{MW}} \leftrightarrow {\text{M}}}}$ and $k_{{{\text{IEW}} \leftrightarrow {\text{NM}}}}$ was not accurately determined due to strong anticorrelation of the fitting results (Supplementary Figure S3).

The longitudinal relaxation rates of the aqueous pools were considerably smaller than those of the nonaqueous pools. Moreover, ${R}_{1}^{\text{MW}}$ exceeded ${R}_{1}^{\text{IEW}}$ by a factor of 2.4 (1.26 ± 0.02 s^−1^ vs. 0.52 ± 0.01 s^−1^), whereas ${R}_{1}^{\text{M}}$ and ${R}_{1}^{\text{NM}}$ were more similar to each other (3.38 ± 0.10 s^−1^ and 2.67 ± 0.14 s^−1^, respectively). As expected, transverse relaxation was faster for MW compared to IEW (factor of 3.3), with ${R}_{2}^{\text{MW}}$=26.85 ± 0.10 s^−1^ and ${R}_{2}^{\text{IEW}}$=8.19 ± 0.03 s^−1^ (i.e., ${T}_{2}^{\text{MW}}$=37 ms and ${T}_{2}^{\text{IEW}}$=122 ms). For the two semisolid pools, the parameter ${T}_{2}$ was largely consistent between pools and across temperatures with an average of 13.3 ± 0.6 µs (assuming a super-Lorentzian lineshape).

### Comparison with results obtained with simpler models

**Fig. 5:**
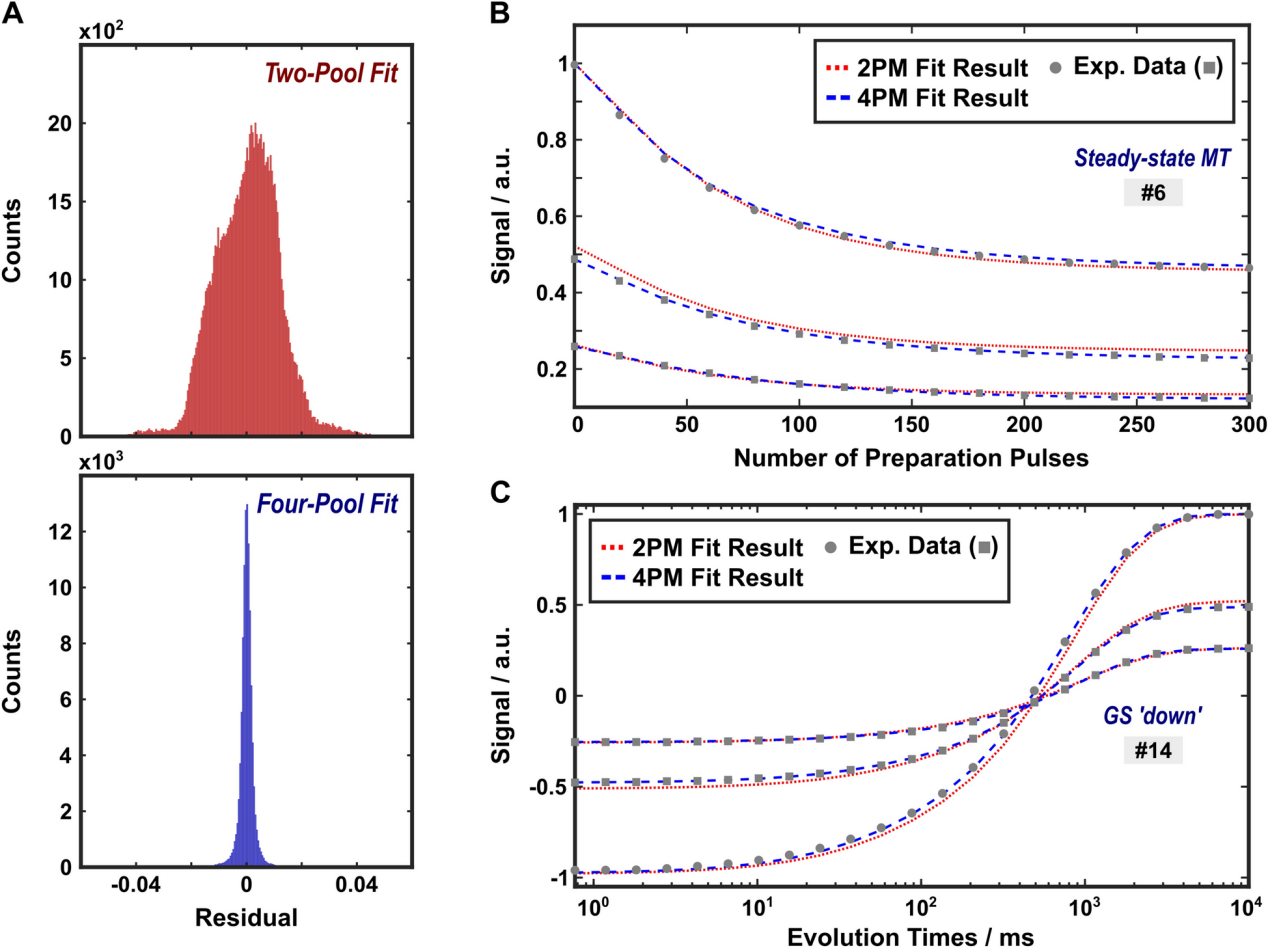
Comparison of the results obtained with fits to the 2PM and the 4PM. (**A**) Histograms of the residuals from a total of 106,320 data points acquired with the 22 protocols (Table 1) at three temperatures. Mean values plus/minus one standard deviation were 0.000386 ± 0.0118 (2PM, red) and − 0.000021 ± 0.0028 (4PM, blue). Systematic improvements obtained with the 4PM were particularly evident for MT-prepared acquisitions and for GS-experiments, which is exemplarily shown for protocols 6 (**B**) and 14 (**C**), respectively.

The same experimental data was also fitted to the simplified 2PM (also expanded by a non-exchanging BW pool; see Supplementary Methods, Eq. S4). The results are summarized in Supplementary Table S4. The agreement between the fit and the experimental data was worse compared to the 4PM, as reflected in increased residuals (Fig. 5A). Such differences were particularly evident for GS experiments and MT-prepared acquisitions (Figs. 5B,C).

The water and semisolid pool fractions estimated with the 2PM, ${A}_{c}^{A}$=0.764 ± 0.004 and ${A}_{c}^{B}$=0.236 ± 0.004, respectively, agreed well with the corresponding combined 4PM fractions ${A}_{c}^{\text{IEW}}+{A}_{c}^{\text{MW}}$=0.773 ± 0.006 and ${A}_{c}^{\text{NM}}+{A}_{c}^{\text{M}}$=0.226 ± 0.003. This demonstrates that the 2PM is not a *specific* measure of myelination although it may correlate with myelin content. A good agreement was also obtained for the line-broadening parameter ${T}_{2}^{B}$ compared to ${T}_{2}^{\text{NM}}$ and ${T}_{2}^{\text{M}}$. Note that the 2PM inherently assumes fast exchange between the IEW and MW pools. Therefore, ${R}_{1}^{A}$=0.81 ± 0.02 s^−1^ and ${R}_{2}^{A}$=15.30 ± 0.05 s^−1^ (at 35 °C) should be compared to weighted average rates of the 4PM aqueous pools, $\langle {R}_{\text{1,2}}^{\text{W}}\rangle =\left({A}_{c}^{\text{IEW}}{R}_{\text{1,2}}^{\text{IEW}}+{A}_{c}^{\text{MW}}{R}_{\text{1,2}}^{\text{MW}}\right)/\left({A}_{c}^{\text{IEW}}+{A}_{c}^{\text{MW}}\right)$, which only yields moderate agreements, $\langle {R}_{1}^{\text{W}}\rangle$=0.93 ± 0.01 s^–1^ and $\langle {R}_{2}^{\text{W}}\rangle$=18.66 ± 0.10 s^–1^. Relevant deviations from a fast-exchange assumption are therefore to be expected not only for transverse but also for longitudinal relaxation.

A notable feature of the CMPG echo train acquired at a an inversion time (${\text{TI}}$) that approximates the zero-crossing time of the inversion-prepared signal (protocol 1) is a transient decay of the echo amplitude towards a minimum and subsequent increase with increasing echo time (${\text{TE}}$) (see Figs. 3A,B). Auxiliary simulations based on the fitting results in Table 1 show that this behavior is a direct consequence of the ${T}_{1}$ difference for the aqueous pools, and would also occur in the absence of exchange (Supplementary Figure S5). The IR preparation of the CPMG readout thus reveals an inadequacy of a simplified model of two water pools that assumes distinct transverse but uniform longitudinal relaxation, which is routinely used for the analysis of multiexponential ${T}_{2}$ experiments.

### Temperature dependence

${k}_{{\text{IEW}}\leftrightarrow {\text{MW}}}(T)$ And ${k}_{{\text{IEW}}\leftrightarrow {\text{NM}}}(T)$ showed Arrhenius behavior with more than fourfold higher activation energy, ${E}_{a}$, for ${k}_{{\text{IEW}}\leftrightarrow {\text{MW}}}$ (29.4 ± 5.0 kJ/mol vs. 6.8 ± 2.9 kJ/mol) whereas ${k}_{{\text{MW}}\leftrightarrow {\text{M}}}(T)$ yielded no robust fit. Results from all analyses of temperature-dependent exchange and relaxation rates are summarized in Supplementary Table S5 and Supplementary Figure S6. The variation of the longitudinal relaxation rates of both aqueous pools was consistent with the assumption of dipolar relaxation in the regime of short correlation times ${\tau }_{c}$ (i.e., ${\omega }_{0}{\tau }_{c}\ll 1$, ${\omega }_{0}$ is the Larmor frequency) and ${R}_{1}\propto {\tau }_{c}$^42^, yielding ${E}_{a}$=10.4 ± 0.8 kJ/mol for IEW whereas the estimate for MW was less accurate (${E}_{a}$=6.0 ± 3.7 kJ/mol). Remarkably, ${R}_{2}$ increased with $T$ for both aqueous pools with estimates of ${E}_{a}$=8.0 ± 0.5 kJ/mol and $6$.2 ± 1.9 kJ/mol, indicating a different dynamic process than the one extracted from the ${R}_{1}$ data. Due to the weaker correlations for the relaxation rates of the nonaqueous pools, estimations of activation energies were not considered meaningful.

### 4PM analyses of ${\varvec{z}}$-spectra

**Fig. 6:**
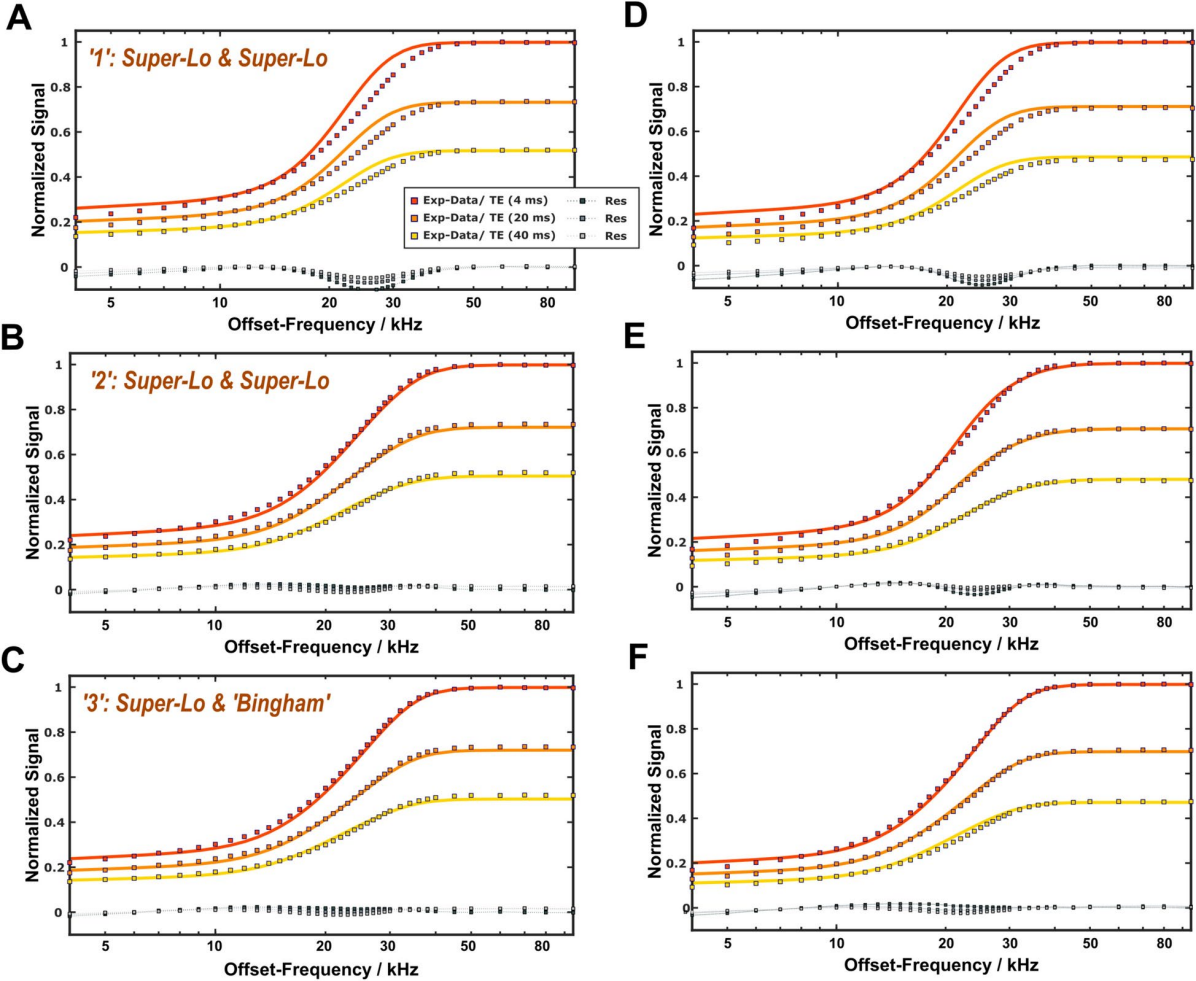
Examples of $z$-spectra acquired with cosine-modulated MT preparation ($\Delta \nu$>4 kHz). Shown are data acquired at 21 °C (**A–C**) and 35 °C (**D–F**) with different TE. A forward simulation using the fitted 4PM parameters from the relaxometry experiments (Table 1) and super-Lorentzian lineshapes for both nonaqueous pools reproduced the experimental data (red, orange and yellow squares) with noticeable residuals at frequencies between 15 and 35 kHz (**A**, **D**). A relevant improvement was achieved after ${T}_{2}^{\text{NM}}$, ${T}_{2}^{\text{M}}$ and ${k}_{{\text{MW}}\leftrightarrow {\text{M}}}$ were adjusted as free parameters (**B**, **E**). Further subtle improvements resulted with a Bingham lineshape for the nonaqueous myelin pool (**C**, **F**) with remaining residuals < 3%, in particular around 20 kHz.

Examples of $z$-spectrum analyses are shown in Fig. 6. Forward simulations (Approach 1) employing the fitted parameters from the relaxometry experiments (Table 1) resulted in systematic underestimations of the saturation of the semisolid pools with corresponding residuals in the range of offset frequencies $\Delta \nu$≈15–35 kHz (Figs. 6A,D). Substantial improvement was achieved by readjusting ${T}_{2}^{\text{NM}}$, ${T}_{2}^{\text{M}}$ and ${k}_{{\text{MW}}\leftrightarrow {\text{M}}}$ to refine the z-spectra fits while keeping all other parameters fixed (Figs. 6B,E). Super-Lorentzian lineshapes^43^ were assumed for both nonaqueous pools in this analysis (Approach 2). The fitted parameters are shown in Supplementary Table S6. Another small improvement was achieved with Approach 3 in which the approximately cylindrical geometry of myelinated axons was taken into account in the dipolar lineshape of the nonaqueous myelin pool (Figs. 6C,F)^25^. In the following, we refer to this lineshape as “Bingham lineshape”.

**Fig. 7:**
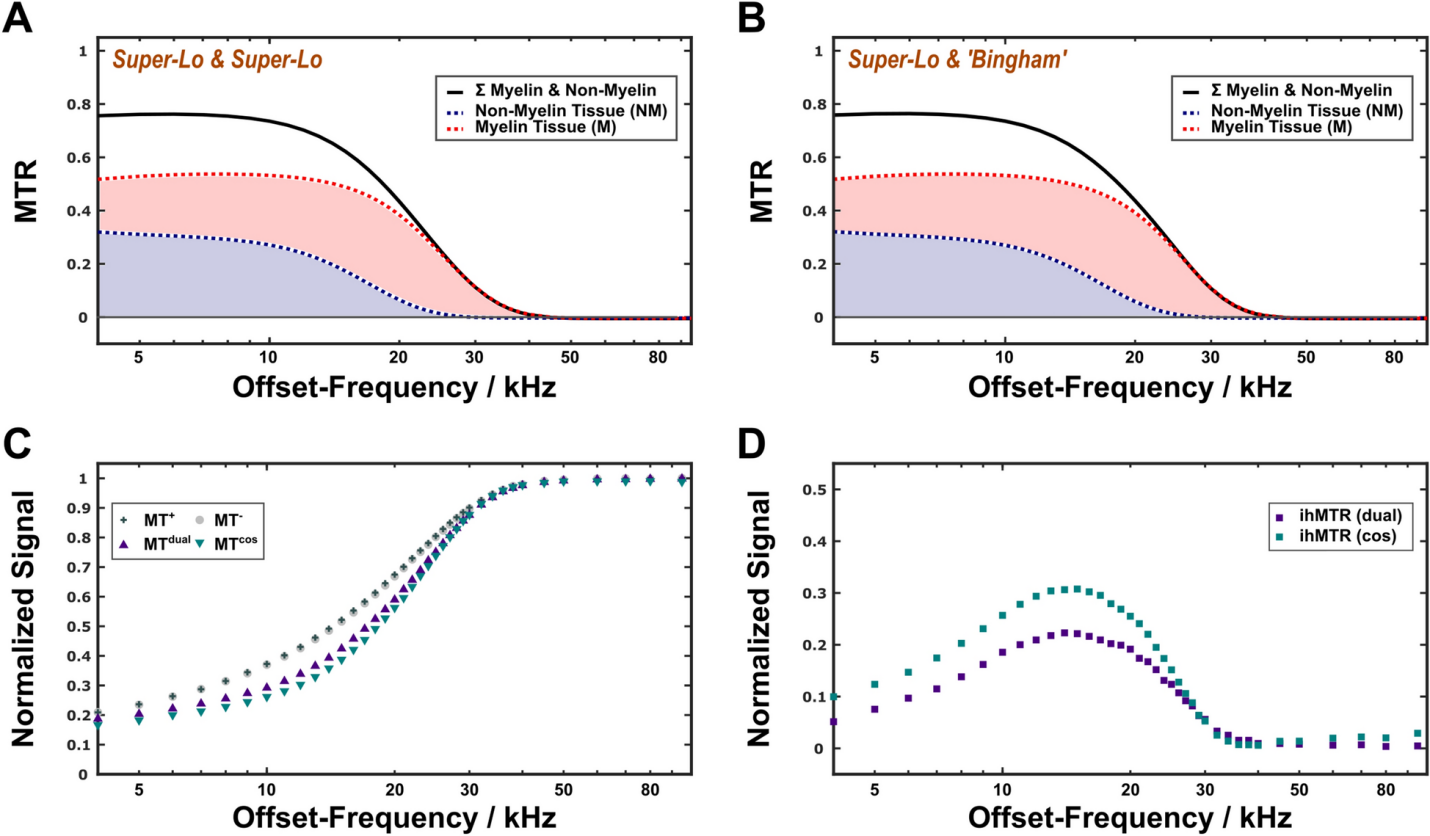
Offset-dependence of the MTR and ihMTR. The total MTR (black solid line) is decomposed into contributions from the nonaqueous non-myelin (blue) and myelin (red) pools. The curves were obtained with forward simulations using the parameters in Supplementary Table S6 for 35 °C and the assumption of super-Lorentzian lineshapes for both pools (**A**) or a super-Lorentizian lineshape ${g}_{sL}^{\text{NM}}$ for pool NM and a Bingham lineshape ${g}_{B}^{\text{M}}$ for pool M (**B**). Datasets of all types of MT preparations (at 34.7 °C only), including also ‘single’-sided off-resonant irradiation (**C**) and the resulting ihMTR (**D**) are also shown. Maximum ihMTRs are on the order of 0.3 for cosine-modulation and 0.2 for dual-sided saturation achieved by offset-frequency alternation.

These results were further used to compare the specific MT effects associated with the two nonaqueous pools (Figs. 7A,B). An MT ratio (${\text{MTR}}$)^16^ was calculated using the parameters in Table 1, except for ${T}_{2}^{\text{NM}}$, ${T}_{2}^{\text{M}}$ and ${k}_{{\text{MW}}\leftrightarrow {\text{M}}}$, which were set to the readjusted values from the $z$-spectrum fits (Supplementary Table S6). Individual contributions were estimated by setting either ${k}_{{\text{MW}}\leftrightarrow {\text{M}}}$=0 or ${k}_{{\text{IEW}}\leftrightarrow {\text{N}}{\text{M}}}$=0 (i.e., MT only within the non-myelin or the myelin compartment, respectively). For $\Delta \nu$<15 kHz, the total ${\text{MTR}}$ was ≈0.75 with individual ratios ${\text{MTR}}^{\text{NM}}$≈0.3 and ${\text{MTR}}^{\text{M}}$≈0.5. Above 15 kHz, the non-myelin-associated ${\text{MTR}}$ decreased more rapidly than the myelin-associated ${\text{MTR}}$ when assuming super-Lorentzian lineshapes for both compartments. This is due to the longer ${T}_{2}^{\text{NM}}$ (16.7 μs vs. 11.3 μs). The same trend was obtained with a Bingham lineshape for the myelin pool. Thus, myelin appears to provide the dominant ${\text{MTR}}$ contribution, albeit without high specificity. The saturation achieved with steady-state MT preparations is shown in Fig. 7C. The inhomogeneous MT-ratio (ihMTR)^44,45^ increased with increasing offset towards maxima (at $\Delta \nu$≈15 kHz) of ≈0.3 for MT-preparation with cosine-modulated pulses (MT^cos^) and ≈0.2 for an MT-preparation scheme with alternating irradiation at positive and negative offset frequencies (MT^±^) (Fig. 7D). For $\Delta \nu$>35 kHz, no MT effect and thus no ihMT effect was detectable.

## Discussion

Standard analysis of relaxometry or MT data of WM in vivo is typically based on a 2PM or simpler models due to the large number of fitting parameters for models of higher complexity^46^. Nevertheless, the presence of at least two exchanging water pools with associated macromolecular pools is well established experimentally^2,28^, and the presence of at least two aqueous pools in vivo is evident from multiexponential ${R}_{2}$ analysis^19–21^. The inadequacy of the 2PM to describe more comprehensive experiments becomes evident at high SNR and is reflected in the current work by substantially increased residuals, as well as systematic deviations for GS- and MT-prepared acquisitions (Fig. 5).

The current work extends the range of earlier measurements^5,18,28^ and yields improved sensitivity to all parameters. In particular, the inclusion of variable MT-preparations yielded additional variation in the initial conditions for both nonaqueous pools, while the CPMG readout helped to separate dipolar relaxation from exchange effects for both water pools. As a result, it was not necessary to assume a fixed ${T}_{1}$ of the nonaqueous pools, which has been typically set to 1 s^22,28^, although recent work suggests considerably faster relaxation^4,6^. Our estimations of ${T}_{1}^{{\text{N}}{\text{M}}}$=374 ± 19 ms and ${T}_{1}^{\text{M}}$=296 ± 9 ms (at 35 °C) can be interpreted as intrinsic relaxation times, which goes beyond an eigenvector approach^5^.

An important finding for the aqueous pools is a relevant difference not only in their ${T}_{2}$ but also in their ${T}_{1}$ times (${T}_{1}^{{\text{M}}{\text{W}}}$=794 ± 13 ms and ${T}_{1}^{\text{IEW}}$=1.92 ± 0.04 s at 35 °C), in contrast to the simplifying assumption ${T}_{1}^{\text{MW}}\approx {T}_{1}^{\text{IEW}}$^5^. Distinct longitudinal relaxation times for IEW and MW are quite plausible given their different microstructural environments^5^. In the current work, a relevant ${T}_{1}$ difference is directly indicated by the transient minimum of the TE-dependent echo amplitude in inversion-prepared CPMG echo trains in the TI region around the zero crossing of the longitudinal magnetization (Fig. 3A,B and Supplementary Figure S5). Since ${R}_{1}^{{\text{M}}{\text{W}}}>{R}_{1}^{\text{IEW}}$, both echo contributions add with a 180° phase difference at TIs, where the faster relaxing ${M}_{z}^{\text{MW}}$ is already positive while the slower relaxing ${M}_{z}^{\text{IEW}}$ is still negative. Due to this ‘antiphase magnetization’ a decay/growth behavior of the echo amplitude is obtained with a characteristic transient minimum. Our result of ${R}_{1}^{\text{MW}}/{R}_{1}^{\text{IEW}}$≈2.2 (at 21 °C) agrees well with the ratio of 2.1 measured by Stanisz et al.^28^ in bovine optic nerve at 20 °C. Other estimations for separate aqueous pools yielded shorter values ranging from ${T}_{1}^{\text{MW}}$≈111–225 ms and ${T}_{1}^{\text{IEW}}$≈1.01–1.15 s at 3 T in vivo^3^ to ${T}_{1}^{\text{MW}}$≈540 ms and ${T}_{1}^{\text{IEW}}$≈740 ms at 1.5 T and room temperature in unfixed porcine and bovine WM obtained 2–4 h *post mortem*^47^. However, a direct comparison of the values is hampered by the fact that tissue fixation required in the current work leads to shifts in the relaxation rates and pool fractions^37,38^. Another contribution to the deviation results from exchange between the water pools, which was not taken into account in the in vivo study.

A separation of compartment-specific relaxation times in multiexponential relaxation experiments requires that mixing of the water pools, which occurs at a rate ${\tau }_{m}^{-1}={k}_{\text{MW,IEW}}+{k}_{\text{IEW,MW}}={k}_{{\text{IEW}}\leftrightarrow {\text{MW}}}\left({A}^{\text{IEW}}+{A}^{\text{MW}}\right),$ is sufficiently slow compared to the shutter speed ${\tau }_{\text{1,2}}^{-1}=\left|{R}_{\text{1,2}}^{\text{IEW}}-{R}_{\text{1,2}}^{\text{MW}}\right|$^12^. The data in Table 1 yield mixing times decreasing from ${\tau }_{m}$≈1.2 s at 21 °C to ≈0.7 s at 35 °C, whereas the shutter speeds were largely temperature independent (${\tau }_{1}$≈1.35 s and ${\tau }_{2}$≈54 ms). Mixing of the two water pools is therefore slow (${\tau }_{m}\gg {\tau }_{2}$) on the ${T}_{2}$ time scale but occurs in an intermediate range (${\tau }_{m}\approx {\tau }_{1}$) on the ${T}_{1}$ time scale. Similar conditions were previously estimated for fixed thalamus and *corpus callosum* samples^48^.

The myelin water fraction, ${\text{MWF}}={A}_{c}^{\text{MW}}/\left({A}_{c}^{\text{IEW}}+{A}_{c}^{\text{MW}}\right)$=0.560 ± 0.004, agreed well with the fraction of 0.57–0.58 of the short-${T}_{2}$ component obtained with biexponential analyses of the CPMG decays (Supplementary Figure S7), whereas biexponentially fitted ${T}_{2}^{\text{IEW}}$ and ${T}_{2}^{\text{MW}}$ values were shorter than the 4PM results (31 ms vs. 37 ms and 102 ms vs. 122 ms at 35 °C). This systematic difference is due to the nonaqueous pools, which are ignored in the multiexponential analysis. Although only longitudinal magnetization exchange between aqueous and nonaqueous pools was directly considered (see Eqs. 2–4), the finite duration of the refocusing pulses (${\tau }_{P}$=200 µs) allows sufficient coupling of transverse and longitudinal aqueous magnetization. This generates a pathway where MT between aqueous and nonaqueous pools contributes to the CMPG decay. Simulations confirmed that our matrix-exponential implementation of the 4PM accounts for this additional signal loss (results not shown). Similar underestimations of relaxation times are expected for published results from multiexponential ${R}_{2}$ analyses, whereas the ${\text{MWF}}$ results should be more robust.

Our MWF estimate is somewhat higher than previously reported values (0.32–0.43) in fixed human or rat spinal cord^37,38^. These works demonstrated substantial increases in the MWF (order of 40%) during fixation. It appears that this leads to an increased absolute size of the MW pool, which is likely related to partial delamination and formation of vacuoles in the myelin sheath. This effect is further amplified in the MWF by a disproportionally larger fixation-related shrinkage of the non-myelin compartment and thus of the IEW pool. Notably, degenerated myelin (myelin debris) appeared to be indistinguishable from intact myelin in relaxometry experiments^38^.

In 2PM analyses, the ‘macromolecular pool fraction’ (${\text{MPF}}={A}_{c}^{B}$ in Supplementary Table S4) is typically considered a measure of myelin content due to strong correlations with histological myelin staining^49^. However, non-zero intercepts of corresponding regression lines indicate the presence of a non-myelin contribution^6^. Similarly, myelin water alone could not explain a ‘nonfreezing’ pool of ‘bound water’ in diffusion experiments in *corpus callosum*^50^, leaving a portion of 31% that was assigned to interfacial water associated with other structures. Remarkably, our finding of a non-myelin contribution of roughly 20% to the semisolid pool in spinal cord WM is of similar magnitude. This underlines that the ${\text{MPF}}$ is not uniquely specific to myelination. Table 1 yields ${\text{MPF}}={A}_{c}^{\text{NM}}+{A}_{c}^{\text{M}}$=0.226 ± 0.003, which compares well with 4PM results of 0.1822–0.298^5^, 0.22^18^, or 0.175–0.187 in bovine WM^2^. However, these studies assumed equal sizes of both nonaqueous pools, which is unlikely given variable myelination in different brain regions^51^.

The temperature dependence of the results permits plausibility checks and the assignment of selected relaxation and exchange rates to specific molecular-dynamic processes. Activation energies from ${T}_{1}$ measurements in purified water are 14.8–15.8 kJ/mol^52,53^, reflecting predominantly rotational Brownian motion of water molecules, which occurs on a ≈2 ps time scale^54^. In biological systems, there is evidence of two intracellular water populations comprising a major fraction with bulk-like dynamics and a smaller fraction (10–15%), which is attributed to the shell of hydration water on macromolecules or interfaces^54–56^. This dynamically perturbed hydration water with longer (rotational) correlation times makes a strong contribution to longitudinal relaxation increasing ${R}_{1}$ over the bulk-water value. Due to rapid exchange between the bulk and hydration shell environments, they cannot be discriminated at typical MRI frequencies, leading to an averaged correlation time^54,57^. The corresponding ${E}_{a}$=9.00 ± 0.42 kJ/mol measured with quasielastic neutron scattering in red blood cells^56^ is close to our result of ${E}_{A}=$ 10.4 ± 0.8 kJ/mol, supporting the assumption of an intrinsic relaxation rate without substantial bias from MT. A larger deviation results for ${R}_{1}^{\text{MW}}$ (${E}_{a}$=6.0 ± 3.7 kJ/mol) and might reflect stronger dynamic retardation if the water is trapped between myelin lamellae. Similar activation energies of 6.5 ± 1.0 and 8.3 ± 3.0 kJ/mol were obtained for a slowly diffusing interfacial water fraction in fixed human^58^ and fresh pig *corpus callosum*, respectively^50^. The average energy required to “break” a hydrogen bond in a locally structured domain is assumed to be 6.3 ± 2.1 kJ/mol^59^. Taken together, this suggests that ${R}_{1}^{\text{MW}}$ is sensitive to translational motion of clusters of water near lipid bilayer head groups or hydrophilic domains of myelin proteins. Estimates of room-temperature correlation times with Eqs. 13 and 14 and a proton-proton distance $a$=151.5 pm^60^ are ${\tau }_{c}$≈9 ps for ${R}_{1}^{\text{IEW}}$ and ${\tau }_{c}$≈19 ps for ${R}_{1}^{\text{MW}}$ and also within the expected range.

Both ${R}_{2}^{\text{IEW}}$ and ${R}_{2}^{\text{MW}}$ *increased* with temperature, in line with temperature dependencies of CPMG decays in *corpus callosum*^50^, peripheral nerve^61^, or muscle^62,63^. An inverse Arrhenius behavior may arise from diffusion in magnetic field gradients at macromolecular interfaces or from (chemical) exchange between water and macromolecules. Although transverse nonaqueous magnetization is neglected in the 4PM, such processes are indirectly accounted for as loss of transverse aqueous magnetization. Both involve hydrogen bonds between water and polar groups on the interface. Consistent with these assumptions, the activation energies of 8.0 ± 0.5 and 6.2 ± 1.9 kJ/mol (Supplementary Table S5) agree well with ${E}_{a}$=6.8 ± 2.9 kJ/mol for ${k}_{{\text{IEW}}\leftrightarrow {\text{NM}}}$ representing MT between the IEW and its associated nonaqueous pool, and also with the energy of 6.3 ± 2.1 kJ/mol for the rearrangement of a hydrogen bond^59^. Consequently, we assume that the temperature dependencies of ${R}_{2}^{\text{IEW}},$ ${R}_{2}^{\text{MW}}$ and ${k}_{{\text{IEW}}\leftrightarrow {\text{NM}}}$ reflect a common dynamic process, probably proton exchange between water and macromolecules. This indicates that MT results primarily from chemical exchange (i.e., between water protons and exchangeable protons of myelin lamellae or other cell membranes) rather than cross-relaxation between water and membrane protons. This is in line with recent research suggesting a dominant contribution from chemical exchange^64^. Both processes, chemical exchange and dipolar cross-relaxation, involve the formation and rearrangement of hydrogen bonds between water molecules and lipid head groups, which is much faster (picosecond to nanosecond timescale) than diffusive intercompartmental water exchange^54^, although slow enough to make a relevant contribution to spin relaxation.

Intercompartmental water exchange is mediated through passive diffusion of water molecules between the inter-/extracellular compartment and the myelin compartment. The estimated ${E}_{a}$ of 29.4 ± 5.0 kJ/mol from ${k}_{{\text{IEW}}\leftrightarrow {\text{MW}}}$ is substantially larger compared with 18.8 kJ/mol reported for self-diffusion in pure water^65^ or values in WM^50^, approaching values observed for diffusion-controlled permeation across phospholipid bilayers of 32.6–53.6 kJ/mol^66^. Therefore, we attribute this activation energy to the myelin membrane’s water permeability, suggesting that the water exchange between the two aqueous pools primarily involves successive permeation of myelin lamellae^9^. It should be noted that transmembrane water exchange in PFA-fixed tissue may be increased compared to the *in-vivo* situation^67^.

A remaining issue of the current 4PM approach is the choice of appropriate lineshapes for the nonaqueous pools. This was irrelevant for the experiments summarized in Supplementary Tables S1 and Fig. 1, which employed only a single offset frequency. The combined analysis of relaxometry data and $z$-spectra demonstrated that the assumption of super-Lorentzians for both nonaqueous pools was inadequate. The lineshape for motional restricted protons represents the sum over all time-averaged dipolar interactions, which cannot easily be captured by a specific mathematical form^2^. Direct measurements of the nonaqueous myelin protons suggest that ≈85% of the signal components are characterized by transverse decay times < 100 µs with a dominant contribution from alkyl chain methylene protons^68,69^. This difficulty is compounded when more than one nonaqueous pool must be considered. Nevertheless, a refinement was achieved by readjustment of selected model parameters (Fig. 6). Although these results were biased by parameter correlations, they indicate different broadenings (i.e., different ${T}_{2}$ s) for the semisolid pools. The previous observation of different dipolar relaxation times ${T}_{1D}$ in WM^70,71^ corroborates this result. The component with the shorter ${T}_{2}$ can be also characterized by a Bingham lineshape with almost identical precision and represents the nonaqueous myelin pool. This assignment is supported by the broad maximum of the ihMTR at offsets of 15–20 kHz, where the MTR difference of both components is maximal (Figs. 7A,D), in line with observations of a stronger correlation of the ihMTR with myelination compared to the MTR^44,72^.

This work confirms that a sophisticated combination of spin-system preparations enables more detailed quantitative assessments of WM microstructure beyond the use of the 4PM as a conceptual basis only^18^. Employing almost unconstrained fitting, the model provided descriptions of a range of relaxometry experiments with unprecedentedly low residuals. This leads to a better understanding of MRI contrast mechanisms and their relationship to elements of tissue composition and microstructure, such as myelination. The developed algorithms and extensive set of quantitative parameters can be used in future simulations of tissue models. This helps to identify the limitations of simpler models, such as the standard 2PM used for steady-state MT experiments or multiexponential ${T}_{1}$ or ${T}_{2}$ analyses, and to evaluate the validity of simplifying assumptions. The integration of realistic lineshape functions into the framework is an obvious step towards further refinement. Similarly, two additional dipolar reservoirs have to be added to account for ihMT effects, which has been circumvented in the current work by restricting the acquisitions to dual-sided excitations. A straightforward implementation is possible, but at the cost of a further increase in the already large number of model parameters and greater mathematical complexity. On the experimental end, orientation-dependent measurements should help to disentangle the superposition of broad absorptions lines of the semi-solid pools, which are characterized by specific geometries of the microstructural elements^25,73^. Clearly, experiments as presented here are not feasible in vivo and even less so in clinical settings due to limitations in scan time and hardware (e.g., unsuitable human-sized volume coils for transmitting hard RF pulses), lower SNR, or the inability of change experimental parameters, such as temperature or sample orientation. However, it is conceivable that a well-chosen subset of measurements can be performed within about 30 min to obtain estimates of selected 4PM parameters that go beyond current “quantitative MRI” approaches.

## Materials and methods

### Sample preparation

The sample was prepared following previously established procedures^73^. Briefly, a piece of porcine spine was obtained from a local slaughterhouse within 2–3 h of death and immediately fixated in 4% PFA in phosphate-buffered saline (PBS) at pH 7.4 at 4 °C. The fixative was changed twice after one and two weeks. After 6 weeks of fixation, the sample was transferred into PBS with 0.1% NaN_3_ until further use. For the MRI experiments, the spinal cord was extracted from the lumbar segment (L5-6), and a WM piece (approx. 17 mm long, 4 mm thick) of the posterior funiculus was excised and washed in PBS for several hours. Subsequently, the specimen was positioned in a 5 mm Wilmad® glass tube (Wilmad-Labglass, NJ, USA) and embedded in Fomblin® (Solvay Solexis, Bollate, Italy) for the MRI measurements. The sample was then stored in PBS/NaN_3_ for approximately 3 months. Multiexponential ${T}_{2}$ and DWI verified the consistency of the MRI parameters with the pre-storage results before performing TEM.

### Transmission electron microscopy

For TEM imaging, 2-mm thick sections of the sample were prepared using a razor blade and post-fixated for 48 h in 2% PFA and 2% glutaraldehyde in 0.2m cacodylate buffer at pH 7.4. Small transverse sections were cut out and contrasted in 1% OsO_4_ in cacodylate buffer for 1 h at room temperature on a laboratory shaker (70 rpm; Rotamax, Heidolph, Schwabach, Germany), followed by two rinses in cacodylate buffer (> 1 h, each) and overnight storage at 4 °C. Dehydration was carried out through a series of acetone solutions (30% for 15 min, 50% for 30 min, 70% for rinsing). The final contrast-enhancement step used 1% uranyl acetate in 70% acetone for 45 min (protected from light), followed by rinsing in 90% acetone on a shaker (30 min, 70 rpm) and in 100% anhydrous acetone (twice for 30 min). The samples were embedded in Durcupan™ araldite resin (Sigma-Aldrich, Steinheim, Germany), and 0.5µm semithin sections were stained with Toluidine Blue (Merck, Darmstadt, Germany) and imaged using a slide scanner (AxioScan Z1; Zeiss, Oberkochen, Germany). For quality control and orientation, light-microscopy images of the stained semithin sections were acquired with a 40 × Zeiss Objective (NA 0.95; ≈300nm resolution) using Zeiss Zen Blue 2.6 microscopy software.

For large field-of-view (FOV) TEM, 50nm sections were prepared with a Reichert Ultracut S (Leica, Wien, Austria) and mounted on slot grids (SF 162 N5 Provac, type 1 × 2 mm^2^) coated with a 10–20nm formvar film. Images were acquired at 80 kV at 3150 × magnification and 4.33nm/px resolution on a Zeiss LEO EM 912 Omega TEM with a 2K-on-axis CCD camera-based YAG scintillator with the software Image SP (TRS-Tröndle, 4nm resolution limit) as a grid of tiles (2048 × 2048) with 15% overlap. 144 tiles in total were acquired per image in 12 × 12 lines from left to right and top to bottom, aligned, adjusted for systematic gray-value differences, and stitched into one image of approximately 75 × 70 µm^2^. Because of differences in stitching and tissue movement during acquisition of the individual tiles, slight variations appeared in the final image size.

### MRI experiments

All MRI experiments were carried out at 3 T on a MAGNETOM Sykra^fit^ (Siemens Healthineers, Erlangen, Germany) using a previously described transmit/receive Helmholtz coil (loop radius and spacing 16 mm)^74^ inside a custom-made temperature control unit^73^. Designed specifically for hour-long studies of small samples, the setup achieves stable measurements with a high signal-to-noise ratio (SNR) and effectively suppresses radiation damping. A fiber-optic system (Optocon; Weidmann, Dresden, Germany) was used for continuous temperature monitoring (Supplementary Figure S8). The measurements were performed at three temperatures and an orientation corresponding to a fiber-to-field angle of ${\theta }_{\text{FB}}$=70°, which was not changed during the scanning session of approximately 10 h. While the choice of a particular orientation is not relevant for the current analysis, we note that some of the 4PM parameters are (likely) orientation-dependent^7,25,73,75,76^.

Dedicated pulse sequences with a one-dimensional (1D) CPMG readout (0.483 mm nominal resolution; echo spacing, $\Delta\text{TE}$=4 ms) were implemented under *syngo* MR VE11E software (Siemens Healthineers). They allowed for a modular combination of customized sequence building blocks (Fig. 1). First, a non-spatially selective preparation module created a non-equilibrium spin state for each pool. It evolved for a variable time $t$ until a short rectangular 90° pulse (duration, ${\tau }_{p}$=20 μs) generated transverse magnetization, which was refocused as a CPMG train combined with a readout gradient along the axis of the cylindrical sample. A ${90}_{x}^{\text{o}}$–${180}_{y}^{\text{o}}$–${90}_{x}^{\text{o}}$ composite pulse (total duration 200 μs, rectangular sub-pulse duration 200/3 μs) was used to compensate for inhomogeneities of the transmit field^77^. A gradient spoiling scheme was implemented, in which gradients were applied along two alternating directions orthogonal to the readout direction with decreasing amplitudes towards the end of the CPMG train to suppress signals from stimulated echoes^78^.

The preparation blocks were chosen to create an extensive range of different initial magnetization states of all aqueous and non-aqueous pools in order to optimize the sensitivity of the fit^5^.

Two types of inversion pulses were utilized for IR experiments (protocols 1 and 2 in Supplementary Table S1): A hard (${\tau }_{p}$=40 μs) rectangular pulse (“RECT”) and a soft (${\tau }_{p}$=5 ms) adiabatic “BIR-4” pulse^79^ as implemented by the vendor with a maximum amplitude of $\gamma {B}_{1}/\left(2\pi \right)\approx 864 \text{Hz}$. The preparation for transient MT measurements (protocol 3) consisted of a composite (on-resonant) MT pulse (16 rectangular sub-pulses, 6 ms total duration), which is assumed to selectively saturate the semisolid pools^80^. For each of the three preparations, the RF pulse was followed by a short spoiler gradient, and acquisitions with 23 logarithmically spaced evolution times (i.e., inversion times for IR sequences; 770 μs ≤ ${\text{TI}}$≤ 10 s; repetition time, ${\text{TR}}$=13 s) were performed. The inversion times were selected to cover a sufficiently large interval, including short ${\text{TI}}$ s < 100 ms to be sensitive to exchange processes following the preparation.

For steady-state MT acquisitions (protocols 4–12; ${\text{TR}}$=6 s), a train of (on-resonance) Gaussian RF pulses (${\tau }_{p}$=2 ms) was applied before the 90° readout pulse of the CPMG train. They were cosine modulated to obtain two frequency bands at offsets of $\Delta \nu$=  ± 15 kHz, and the amplitude was scaled by a factor of $\sqrt{2}$^72,81^. Use of cosine-modulated pulses allowed to neglect dipolar-order effects^82^. Sixteen acquisitions were made with each protocol, with an incremental increase of the number of preparation pulses, ${N}_{\text{RF}}$, by 20 pulses with each repetition. This incremental approach was used because steady-state MT saturation measurements are only sensitive to the product of multiple spin-system parameters^22^. These experiments were performed with three root-mean-squared transmit amplitudes, $\gamma {B}_{1,\text{RMS}}/\left(2\pi \right)$, of 500 Hz, 750 Hz and 1000 Hz ($\gamma$ is the gyromagnetic ratio) and three inter-pulse delays ${\tau }_{s}$ of 0.25 ms, 2.25 ms and 4.25 ms (Supplementary Table S1).

Goldman–Shen experiments (protocols 13–16 with identical ${\text{TI}}$ s and ${\text{TR}}$ as in protocols 1–3) were performed to separate the two aqueous reservoirs based on their ${T}_{2}$. The sequence was implemented as in previous work with filter-times ${\tau }_{f}$ of 1 and 50 ms^5^. The version with ${\tau }_{f}$=50 ms is expected to separate signal contributions from MW and IEW, while the version with ${\tau }_{f}$=1 ms should roughly separate contributions from the nonaqueous and aqueous magnetization.

To further extend the range of initial conditions of the magnetization, IR experiments (${\text{TI}}$ s and ${\text{TR}}$ as in protocol 1) with a hard inversion pulse were combined with MT preparation. In detail, a train of Gaussian MT pulses (as in protocols 4–12) was applied *before* the inversion pulse to generate different levels of saturation of the nonaqueous pools, and another train of Gaussian MT pulses was applied *after* the inversion pulse during the evolution period whenever $\text{TI}>{\tau }_{s}+{\tau }_{p}$, but was omitted if $\text{TI}\le {\tau }_{s}+{\tau }_{p}$. As in protocols 4–12, different selections of ${\tau }_{s}$ were compared (protocols 17, 19 and 21). Finally, the same experiments were also performed without application of the inversion pulse with otherwise identical parameters (protocols 18, 20, 22).

Besides the relaxometry measurements, $$z$$-spectra were acquired with pulsed MT-preparation followed by a 90° readout pulse and 1D spatial encoding ($${\text{TR}}$$=6 s; 0.483 mm nominal resolution). As described above, Gaussian MT pulses $$({N}_{\text{RF}}$$=300, $${B}_{1,\text{RMS}}$$=500 Hz, $${\tau }_{p}$$=2 ms, $${\tau }_{s}$$=250 μs $$)$$ were employed with 42 offset frequencies ($$\Delta \nu $$=0, 1, 2, …, 30, 32, 34, …, 40, 45, 50, 60, 70, 80, 95 kHz). Four types of saturation were compared: *(i)* single-sided at positive (referred to as $${\text{MT}}^{+}$$) and *(ii)* at negative $$\Delta \nu $$ ($${\text{MT}}^{-}$$), *(iii)* dual-sided with alternating sign of $$\Delta \nu $$ in subsequent RF events ($${\text{MT}}^{\pm }$$), and *(iv)* dual-sided achieved by cosine modulation ($${\text{MT}}^{\text{cos}}$$).

In a separate experiment, DWI was performed at room temperature with a 1D Stejskal-Tanner sequence^83^ with 60 gradient directions interleaved with 7 acquisitions without diffusion-sensitizing gradients (echo time, ${\text{TE}}$=80 ms; ${\text{TR}}$=4 s; *b*-value 1500 s/mm^2^; 0.483 mm nominal resolution).

### Data analysis

The entire data analysis was implemented in Matlab (R2020b; MathWorks, Natick, MA, USA). The raw *k*-space data was transferred from the scanner, multiplied by a Tukey window (cosine fraction $r$= 0.5)^84^, Fourier-transformed and phase-corrected. For non-negative least-squares (NNLS) fitting, standard algorithms from Matlab’s Optimization Toolbox (‘lsqnonlin’) were used.

### Analysis of relaxometry data

The standard 2PM was extended to four exchanging pools as shown in Supplementary Figure S1 with an additional pool BW pool without exchange with any of the other reservoirs^5^. Each pool $l\in \left\{{\text{IEW}}, {\text{NM}}, {\text{MW}}, {\text{M}}, {\text{BW}}\right\}$ is characterized by a fractional size ${A}^{l}={M}_{0}^{l}/{\sum }_{l}{M}_{0}^{l}$ and relaxation rates ${R}_{\text{1,2}}^{l}=1/{T}_{\text{1,2}}^{l}$, where ${M}_{0}^{l}$ is the equilibrium magnetization. By definition, the fractional pool sizes are normalized (i.e., ${\sum }_{l}{A}^{l}$=1). Diffusion processes lead to an exchange of magnetization between MW and IEW, which is characterized by rate constants ${k}_{{\text{IEW}},{\text{MW}}}$ and ${k}_{{\text{MW}},{\text{IEW}}}$ for the forward and backward direction, respectively^2,3,5^. Similarly, MT between the water pools and their associated semisolid pools was characterized by ${k}_{{\text{IEW}},{\text{NM}}}$ and ${k}_{{\text{NM}},{\text{IEW}}}$ as well as ${k}_{\text{MW,M}}$ and ${k}_{\text{M,MW}}$^2^. Since the rate constants are pairwise interrelated by steady-state conditions ${k}_{l,m}{M}_{0}^{l}={k}_{m,l}{M}_{0}^{m}$, they were expressed in terms of fundamental rate constants

1 $$ k_{l \leftrightarrow m} = \frac{{k_{l,m} }}{{A^{m} }} = \frac{{k_{m,l} }}{{A^{l} }}. $$

Note that ${k}_{l\leftrightarrow m}$ differs from the corresponding constant $R$ of Henkelman et al.^22^ by additional normalization, ${k}_{l\leftrightarrow m}=R{\sum }_{l}{M}_{0}^{l}$, which is convenient as only relative pool sizes are obtained from the fits. A temperature-independent scaling factor ${f}_{B1}$ that was applied to all RF pulses was finally fitted to correct for potential adjustment errors (deviations were < 0.6% when ${f}_{B1}$ was treated as temperature dependent). In order to obtain converging fits despite the large number of model parameters ($n$=18), the concatenated data from all relaxometry experiments (Supplementary Table S1) was used in its entity for fitting. This included the acquisitions at different temperatures, that is, the number of free parameters was almost tripled ($n$=44) to account for potential temperature dependence of relaxation and exchange rate constants. The fractional pool sizes were assumed to be temperature-independent. Starting values and lower and upper bounds of the fits are summarized in Supplementary Table S7.

The fitting procedure included a forward calculation of the entire pulse sequence for each experiment based on an established matrix-algebra approach^24,39^. For this calculation, the sequence timing and pulse envelopes were parsed directly from the scanner’s raw data (Twix format) and integrated into the simulation framework analogous to the sequence source code. Relaxation during RF pulses was explicitly considered. Spoiler gradients were assumed to achieve perfect spoiling. Analogous to the treatment of the 2PM^24,73^, the Bloch-McConnell Equations^40,41^ were converted into a homogeneous form^85,86^ to describe the evolution of the magnetization vector $\mathbf{M}$ under the influence of the dynamic matrix $\mathcal{L}$. Briefly, $\mathcal{L}$ takes into account all relaxation and exchange processes and the effects of RF pulses applied with an offset frequency $\Omega =2{\uppi \Delta }\upnu ={\omega }_{0}-{\omega }_{\text{RF}}$ (${\omega }_{0}$ and ${\omega }_{\text{RF}}$ are the Larmor frequency and the RF field’s frequency, respectively) and a (time-dependent) amplitude (in rad/s) $$-\gamma {\mathbf{B}}_{1}={\left(\begin{array}{ccc}{\omega }_{1x}& {\omega }_{1y}& 0\end{array}\right)}^{T}$$:

2 $$ \frac{{d{\mathbf{M}}}}{dt} = - {\mathbf{\mathcal{L}}} \cdot {\text{M}}. $$

As is customary in MT modeling, transverse magnetization of the semisolid pools was neglected due to their very short ${T}_{2}$ times^22,35^, so the total proton magnetization of the 4PM with an additional non-exchanging BW pool is:

3 $$ {\mathbf{M}} = \left( {\begin{array}{*{20}c} \frac{1}{2} & {M_{x}^{{{\text{IEW}}}} } & {M_{y}^{{{\text{IEW}}}} } & {M_{z}^{{{\text{IEW}}}} } & {M_{z}^{{{\text{NM}}}} } & {M_{x}^{{{\text{MW}}}} } & {M_{y}^{{{\text{MW}}}} } & {M_{z}^{{{\text{MW}}}} } & {M_{z}^{{\text{M}}} } & {M_{x}^{{{\text{BW}}}} } & {M_{y}^{{{\text{BW}}}} } & {M_{z}^{{{\text{BW}}}} } \\ \end{array} } \right)^{T} . $$

$\mathcal{L}$ is, therefore, a square matrix of order 12 (see Supplementary Methods; Eqs. S1–S3). In compact form, it can be written as a block matrix:

4 $$ {\mathbf{\mathcal{L}}} = \left( {\begin{array}{*{20}l} 0 \hfill & 0 \hfill & 0 \hfill & 0 \hfill \\ {{{\varvec{\Lambda}}}_{0}^{{{\text{IEW}},{\text{NM}}}} } \hfill & {{{\varvec{\Lambda}}}^{{{\text{IEW}},{\text{NM}}}} + {\mathbf{K}}^{{{\text{IEW}},{\text{MW}}}} } \hfill & { - {\mathbf{K}}^{{{\text{MW}},{\text{IEW}}}} } \hfill & 0 \hfill \\ {{{\varvec{\Lambda}}}_{0}^{{{\text{MW}},{\text{M}}}} } \hfill & { - {\mathbf{K}}^{{{\text{IEW}},{\text{MW}}}} } \hfill & {{{\varvec{\Lambda}}}^{{{\text{MW}},{\text{M}}}} + {\mathbf{K}}^{{{\text{MW}},{\text{IEW}}}} } \hfill & 0 \hfill \\ {{\mathbf{L}}_{0}^{{{\text{BW}}}} } \hfill & 0 \hfill & 0 \hfill & {{\mathbf{L}}_{a}^{{{\text{BW}}}} } \hfill \\ \end{array} } \right). $$

The submatrices

5 $$ {\mathbf{L}}_{0}^{l} = \left( {\begin{array}{*{20}l} 0 \hfill \\ 0 \hfill \\ { - 2R_{1}^{l} M_{0}^{l} } \hfill \\ \end{array} } \right) \; {\text{and}}\; {\mathbf{L}}_{a}^{l} = \left( {\begin{array}{*{20}l} {R_{2}^{l} } \hfill & {\Omega } \hfill & { - \omega_{1y} } \hfill \\ { - {\Omega }} \hfill & {R_{2}^{l} } \hfill & {\omega_{1x} } \hfill \\ {\omega_{1y} } \hfill & { - \omega_{1x} } \hfill & {R_{1}^{l} } \hfill \\ \end{array} } \right)\;{\text{with}}\;l \in \left\{ {\text{IEW, MW, BW}} \right\} $$

represent the Bloch equations for the water pools, and

6 $$ {{\varvec{\Lambda}}}_{0}^{l,m} = \left( {\begin{array}{*{20}l} {{\mathbf{L}}_{0}^{l} } \hfill \\ { - 2R_{1}^{m} M_{0}^{m} } \hfill \\ \end{array} } \right) \; {\text{and}}\; {{\varvec{\Lambda}}}^{l,m} = \left( {\begin{array}{*{20}l} {{\mathbf{L}}_{a}^{l} + {\mathbf{k}}^{l,m} } \hfill & { - \left( {{{\varvec{\upkappa}}}^{m,l} } \right)^{T} } \hfill \\ { - {{\varvec{\upkappa}}}^{l,m} } \hfill & {R_{1}^{m} + k_{m,l} + R_{{{\text{RF}}}}^{m} } \hfill \\ \end{array} } \right)\;{\text{with}}\;\left( {l,m} \right) \in \left\{ {\left( {{\text{IEW}},{\text{nM}}} \right){, }\left( {{\text{MW}},{\text{M}}} \right)} \right\} $$

represent a 2PM with an aqueous pool $l$ and associated nonaqueous pool $m$. Magnetization exchange between two pools $l\ne m$ is described by

7 $$ {\mathbf{k}}^{l,m} = \left( {\begin{array}{*{20}l} {k_{l,m} } \hfill & 0 \hfill & 0 \hfill \\ 0 \hfill & {k_{l,m} } \hfill & 0 \hfill \\ 0 \hfill & 0 \hfill & {k_{l,m} } \hfill \\ \end{array} } \right). $$

In particular, chemical exchange between the tissue water pools is represented by

8 $$ {\mathbf{K}}^{l,m} = \left( {\begin{array}{*{20}c} {{\mathbf{k}}^{l,m} } & 0 \\ 0 & 0 \\ \end{array} } \right) \;{\text{with}}\;l,m \in \left\{ {\text{IEW, MW}} \right\} \wedge l \ne m, $$

and MT between an aqueous and a nonaqueous pool is represented by

9 $$ {{\varvec{\upkappa}}}^{l,m} = \left( {\begin{array}{*{20}c} 0 & 0 & {k_{l,m} } \\ \end{array} } \right) \Rightarrow \left( {{{\varvec{\upkappa}}}^{m,l} } \right)^{T} = \left( {\begin{array}{*{20}c} 0 \\ 0 \\ {k_{m,l} } \\ \end{array} } \right)\;{\text{with}}\;\left( {l,m} \right) \in \left\{ {\left( {{\text{IEW}},{\text{NM}}} \right){, }\left( {{\text{MW}},{\text{M}}} \right)} \right\}. $$

The standard 2PM describes the effect of an RF pulse on the magnetization of the semisolid pools $m\in \left\{\text{NM},\text{M}\right\}$ by means of an RF absorption rate^22,26^

10 $$ R_{{{\text{RF}}}}^{m} = \pi \omega_{1}^{2} g^{m} \left( {{\Omega },T_{2}^{m} } \right), $$

which leads to (partial) saturation. It is calculated assuming a suitable lineshape ${g}^{m}\left(\Omega ,{T}_{2}^{m}\right)$, such as a super-Lorentzian^43^ (see Supplementary Methods, Eq. S5). Note that ${T}_{2}^{m}$ is not genuine relaxation time according to the Bloch equations but characterizes the dipolar broadening of the (non-Lorentzian) absorption line.

If ${\mathcal{L}}^{(i)}$ is time-invariant during an arbitrary interval $\Delta {t}_{i}$, the solution to Eq. 2 is easily obtained as

11 $$ {\mathbf{M}}\left( {t + {\Delta }t_{i} } \right) = \exp \left( { - {\Delta }t_{i} {\mathbf{\mathcal{L}}}^{\left( i \right)} } \right) \cdot {\text{M}}\left( t \right) \equiv {\mathbf{\mathcal{P}}}^{\left( i \right)} \cdot {\text{M}}\left( t \right). $$

The matrix exponential $\text{exp}\left(-\Delta {t}_{i}{\mathcal{L}}^{(i)}\right)$, and hence $\mathbf{M}\left(t+\Delta {t}_{i}\right)$, can be calculated with arbitrary precision using numerical techniques^87^ and can be substantially accelerated by suitable interpolation along the ‘dimension’ of the RF amplitudes^24,39^. The entire evolution of the magnetization was, therefore, obtained by dividing the pulse sequence into $n$ episodes of (individual) duration $\Delta {t}_{i}$, during which all elements of ${\mathcal{L}}^{(i)}$ remained constant, and consecutive application of the corresponding propagator ${\mathcal{P}}^{(i)}$ according to:

12 $$ {\mathbf{M}}\left( {t + \mathop \sum \limits_{i = 1}^{n} {\Delta }t_{i} } \right) = \left( {\mathop \prod \limits_{i = 0}^{n - 1} {\mathbf{\mathcal{P}}}^{\left( i \right)} } \right) \cdot {\mathbf{M}}\left( t \right). $$

However, the assumption that RF irradiation saturates the semisolid pools according to Eq. 10 is not valid for hard on-resonance pulses with ${\gamma }^{2}{B}_{1}^{2}{T}_{1}^{m}{T}_{2}^{m}\gg 1$. In particular, the 40µs inversion pulse in protocol 1 leads to (partial) inversion of ${M}_{z}^{\text{NM}}$ and ${M}_{z}^{\text{M}}$. The effect of on-resonance pulses on ${M}_{z}^{\text{NM}}$ and ${M}_{z}^{\text{M}}$ was therefore determined by directly fitting the elements of the propagator matrix ${\mathcal{P}}^{(i)}$ acting on ${M}_{z}^{\text{nM}}$ and ${M}_{z}^{\text{M}}$ (i.e., ${\mathcal{P}}_{\text{5,5}}^{\left(i\right)}$ and ${\mathcal{P}}_{\text{9,9}}^{\left(i\right)}$) instead of these particular matrix-exponential elements^73^. This corresponds to fitting effective flip angles ${\alpha }_{\text{eff}}^{m}$ for the nonaqueous pools.

### Temperature dependence of the model parameters

The relaxation parameters were further analyzed assuming dipolar relaxation characterized by a single correlation time ${\tau }_{c}$^42,88,89^. If the dominant mechanism results from intramolecular dipole couplings (i.e., a single proton pair separated by a distance $a$), which are modulated by random molecular reorientations, the relaxation rates are given by

13 $$ R_{1} \approx R_{2} \approx \left( {\frac{{\mu_{0} }}{4\pi }} \right)^{2} \frac{3}{2} \cdot \frac{{\gamma^{4} \hbar^{2} }}{{a^{6} }}\tau_{c} { }\;{\text{ for }}\;{ }\omega_{0}^{2} \tau_{c}^{2} \ll 1, $$

where ${\mu }_{0}$ is the magnetic permeability of free space and $\hslash$ the (reduced) Planck constant. The temperature dependencies of ${\tau }_{c}$ and also of ${k}_{l\leftrightarrow m}$ were expressed by Arrhenius Equations^90^:

14 $$ \tau_{c} = \tau_{c}^{\infty } \exp \left( { + \frac{{E_{a} }}{RT}} \right){ }\;{\text{and}}\;k_{l \leftrightarrow m} = k_{l \leftrightarrow m}^{\infty } \exp \left( { - \frac{{E_{a} }}{RT}} \right) $$

with pre-exponential factors ${\tau }_{c}^{\infty }$ and ${k}_{l\leftrightarrow m}^{\infty }$. ${E}_{a}$ is the activation energy, and $R$ the universal gas constant. Since only data from three temperatures were available, fits to linearized Arrhenius plots with squared Pearson correlation coefficients 0.81 ≤ ${r}^{2}$<0.9 were considered as rough estimates and those with ${r}^{2}$≥0.9 as reliable. Other results were discarded.

### Analysis of ${\varvec{z}}$-spectra

The $z$-spectra were analyzed separately from the relaxometry data, but also within the 4PM framework. The following approaches were compared: Forward simulations assuming super-Lorentzian lineshapes for both nonaqueous pools and using the fitted parameters from the relaxometry experiments (‘Approach 1’); refined simulations with fixed parameters as in Approach 1 except for ${T}_{2}^{\text{NM}}$, ${T}_{2}^{\text{M}}$ and ${k}_{{\text{MW}}\leftrightarrow {\text{M}}}$, which were fitted to the $z$-spectra (‘Approach 2’); and fits as in Approach 2, but assuming an alternative lineshape for the nonaqueous myelin pool (‘Approach 3’). It considers a cylindrical symmetry of myelinated axons and a realistic distribution of fiber orientations within the voxel (here, 483µm-wide 1D projections through the cylindrical sample) as described elsewhere^25^. Briefly, to account for multidirectionality, a fiber orientation distribution function (fODF) was estimated by spherical deconvolution from the DWI data^91,92^ and parameterized using a scaled Bingham probability distribution function^93^:

15 $$ \beta \left( {\mathbf{u}} \right) = f_{0} \exp \left[ { - \kappa_{1} \left( {{{\varvec{\upmu}}}_{1} \cdot {\mathbf{u}}} \right)^{2} - \kappa_{2} \left( {{{\varvec{\upmu}}}_{2} \cdot {\mathbf{u}}} \right)^{2} } \right]. $$

${f}_{0}$ is a scaling parameter. The vector $\mathbf{u}$ indicates a point on the unit sphere's surface, and ${\kappa }_{1}$ ≤ ${\kappa }_{2}$ are so-called concentration parameters along axes ${{\varvec{\upmu}}}_{1}$ and ${{\varvec{\upmu}}}_{2}$, respectively, which characterize the width, ovality and orientation of the distribution. The mean direction of the distribution is given by the vector ${{\varvec{\upmu}}}_{0}={{\varvec{\upmu}}}_{1}{\times {\varvec{\upmu}}}_{2}$. In the following, we refer to the resulting lineshape as “Bingham lineshape” ${g}_{B}^{\text{M}}\left(\Omega ,{T}_{2}^{\text{M}}\right)$ (see Supplementary Methods, Eqs. S6–S8).

Additionally, the customary ‘MT-ratio’^16^,

16 $$ {\text{MTR}} = \frac{{S_{0} - S\left( {{\text{MT}}^{{{\text{cos}}}} } \right)}}{{S_{0} }}, $$

and an ’inhomogeneous MT-ratio’^44,45^,

17 $$ {\text{ihMTR}} = \frac{{S\left( {{\text{MT}}^{ + } } \right) + S\left( {{\text{MT}}^{ - } } \right) - 2S\left( {{\text{MT}}^{{{\text{dual}}}} } \right)}}{{2S_{0} }}\;{\text{with}}\;{\text{MT}}^{{{\text{dual}}}} \in \left\{ {{\text{MT}}^{ \pm } ,{\text{MT}}^{{{\text{cos}}}} } \right\}, $$

were also computed. ${S}_{0}$ and $S({\text{MT}})$ with ${\text{MT}} \in \left\{ {{\text{MT}}^{ + } ,{\text{MT}}^{ - } ,{\text{MT}}^{ \pm } ,{\text{MT}}^{{{\text{cos}}}} } \right\}$ denote the signal amplitudes measured without and with MT-preparation, respectively.

### Analysis of diffusion data

Preprocessing and simplifying tensor-based analyses of diffusion-weighted images were done using FSL 4.1.4 (www.fmrib.ox.ac.uk/fsl). For a full description of the procedures to estimate the Bingham parameters, we refer to Refs. 25 and 93. In short, MRtrix (www.brain.org.au/software/mrtrix) was applied for constrained spherical deconvolution, and the resulting fODF was approximated by eighth-order spherical harmonics expansion. A region in the center of the sample was manually selected to compute the single fiber response function. For identifying peaks indicating fiber bundles, the fODF was overlayed with a discrete grid to search for local maxima. Bingham functions were fitted to the largest two maxima, whereas further peaks were considered to be artifacts or noise.

## Supplementary Information

Supplementary Material 1.

## Data Availability

All MR data and code needed for reproducing or extending our analyses are present in the main text and/or the Supplementary Materials will be made available upon acceptance. Pre-processed MR data will be made available upon acceptance at https://dataverse.harvard.edu/ (10.7910/DVN/1JR9DT) or can be obtained from the corresponding author (N. Wallstein). Binary code of the pulse sequences used in the current work can be made available upon written request to the authors for use at other institutions on compatible scanners. Note that pulse sequences implemented with IDEA (Integrated Development Environment for Applications; Siemens Healthineers) contains vendor-specific code. Therefore, sharing sequence code can only take place via the customer-to-customer partnership program (so-called C2P procedure).
