# Supplementary material for: An unconstrained four pool model analysis of proton relaxation and magnetization transfer in ex vivo white matter
Source: Sci Rep. 2025 Feb 5;15:4354. doi: 10.1038/s41598-025-87362-4 (PMC11799436; doi:10.1038/s41598-025-87362-4)
Supplement: Supplementary file 1 — Supplementary Material 1. [file 41598_2025_87362_MOESM1_ESM.pdf]

**Supplementary Material for**

# An Unconstrained Four Pool-Model Analysis of Proton Relaxation and Magnetization Transfer in *Ex Vivo* White Matter

Niklas Wallstein[1], André Pampel[1], Roland Müller[1], Carsten Jäger[2,3], Markus Morawski[3] and Harald E. Möller[1,4]

*[1] NMR Methods & Development Group, Max Planck Institute for Human Cognitive and Brain Sciences, Leipzig, Germany*

*[2] Department of Neurophysics, Max Planck Institute for Human Cognitive and Brain Sciences, Leipzig, Germany*

*[3] Paul Flechsig Institute – Center of Neuropathology and Brain Research, Medical Faculty, Leipzig University, Germany*

*[4] Felix Bloch Institute for Solid State Physics, Leipzig University, Germany*

## Abbreviations

1D = one-dimensional, 2PM = two-pool model, 4PM = four-pool model, BW = bulk water, CPMG = Carr-Purcell-Meiboom-Gill, DWI = diffusion-weighted imaging; fODF = fiber orientation distribution function, FOV = field of view; GS = Goldman-Shen, IEW = intra-/extracellular water, ihMT = inhomogeneous magnetization transfer; IR = inversion recovery, M = non-aqueous myelin, MRI = magnetic resonance imaging, MT = magnetization transfer, $\mathrm{MT}^{+}$ = MT preparation by irradiation at positive offset frequencies, $\mathrm{MT}^{-}$ = MT preparation by irradiation at negative offset frequencies, $\mathrm{MT}^{\pm}$ = MT preparation by alternating irradiation at positive and negative offset frequencies, $\mathrm{MT}^{\cos}$ = MT preparation by cosine-modulated pulses, MW = myelin water, NM = non-aqueous non-myelin, NNLS = non-negative least squares, PBS = phosphate-buffered saline, PFA = paraformaldehyde; RF = radiofrequency, SNR = signal-to-noise ratio, TEM = transmission electron microscopy, WM = white matter.

## Mathematical symbols

| | |
|---|---|
| $A^l$: | fractional size of proton pool $l$, |
| $A_c^l$: | corrected fractional size of proton pool $l$, |
| $a$: | proton-proton distance, |
| $\mathbf{B}_0$: | static magnetic field vector, |
| $\mathbf{B}_1$: | RF magnetic field vector, |
| $B_1$: | RF magnetic field amplitude, |

$B_{1,\mathrm{RMS}}$: root-mean-squared amplitude of the transmit RF magnetic field,
$b$: $b$-factor,
$E_a$: activation energy,
$f_0$: scaling factor,
$f_{B1}$: pulse-amplitude correction factor,
$g_{\mathrm{cyl}}^{\mathrm{M}}$: lineshape function for a single cylindrical fiber,
$g^m$: absorption lineshape function for a non-aqueous pool $m$,
$g_B^m$: Bingham lineshape for a non-aqueous pool $m$,
$g_{sL}^m$: super-Lorentzian lineshape for a non-aqueous pool $m$,
$\hbar$: reduced Planck constant
$i$: integer,
ihMTR: inhomogeneous MT ratio,
$\mathbf{K}^{l,m}$: submatrix of $\boldsymbol{\mathcal{L}}$ describing chemical exchange between water pools $l$ and $m$,
$k$: spatial frequency,
$k_{l,m}$: pseudo-first-order rate constant for unidirectional magnetization transfer from pool $l$ to pool $m$,
$k_{l\leftrightarrow m}$: fundamental rate constant for bidirectional exchange of magnetization between pools $l$ and $m$,
$k_{l\leftrightarrow m}^{\infty}$: pre-exponential factor,
$\mathbf{k}^{l,m}$: submatrix of $\boldsymbol{\mathcal{L}}$ describing exchange of magnetization between pools $l$ and $m$,
$\boldsymbol{\mathcal{k}}$: matrix describing exchange effects,
$\boldsymbol{\mathcal{L}}$: dynamic matrix,
$\boldsymbol{\mathcal{L}}^{(i)}$: $i$-th step of the dynamic matrix, during which it is time-invariant,
$\mathbf{L}_0^l, \mathbf{L}_a^l$: submatrices of $\boldsymbol{\mathcal{L}}$ representing the Bloch equations for water pool $l$,
$l, m$: pool indices,
$\boldsymbol{\ell}$: matrix describing intrinsic relaxation (without exchange) and RF pulse effects,
$\mathbf{M}$: magnetization vector,
$M_0^l$: equilibrium magnetization of pool $l$,
$M_x^l, M_y^l, M_z^l$: Cartesian coordinates of the magnetization of pool $l$,
MPF: macromolecular pool fraction,
MTR: MT ratio,
$N_{\mathrm{RF}}$: number of RF pulses,
$\mathcal{N}$: normalization factor,
$n$: integer,
$\boldsymbol{\mathcal{P}}^{(i)}$: propagator for the $i$-th time interval,
$R$: universal gas constant,
$R_1$: longitudinal relaxation rate,
$R_1^l$: longitudinal relaxation rate of pool $l$,
$R_2^l$: transverse relaxation rate of pool $l$,
$R_2^{\mathrm{res}}$: residual linewidth parameter of a bilayer segment at the magic angle,
$\langle R_{1,2}^{\mathrm{W}}\rangle$: weighted average relaxation rates $R_{1,2}$ of the water pools,
$R_{\mathrm{RF}}^m$: RF absorption rate of macromolecular proton pool $m$,
$r$: cosine fraction of the Tukey window,
$r, r^2$: Pearson correlation coefficient and squared Pearson correlation coefficient,
$r_2^{\mathrm{M}}$: linewidth parameter of a cylindrical fiber,
$S_0$: signal amplitude without MT-preparation,

$S(\mathrm{MT})$: signal amplitude with MT-preparation,
$T$: absolute temperature,
$T_1$: longitudinal relaxation time,
$T_1^l$: longitudinal relaxation time of pool $l$,
$T_{1D}$: dipolar longitudinal relaxation time,
$T_2$: transverse relaxation time,
$T_2^l$: transverse relaxation time of pool $l$,
TE: echo time,
$\Delta$TE: echo spacing,
TI: inversion time,
TR: repetition time,
$t$: time,
$\Delta t_i$: $i$-th time interval,
$\mathbf{u}$: vector to a point on the unit sphere,
$x, y, z$: Cartesian coordinates,
$\alpha_{\mathrm{eff}}^m$: effective flip angle due to an on-resonant RF pulse acting on pool $m$,
$\beta(\mathbf{u})$: scaled Bingham probability distribution,
$\gamma$: gyromagnetic ratio,
$\theta$: polar angle,
$\theta_{\mathrm{FB}}$: fiber-to-field angle,
$\kappa_{1,2}$: Bingham concentration parameters,
$\boldsymbol{\kappa}^{l,m}, (\boldsymbol{\kappa}^{m,l})^T$: submatrices of $\boldsymbol{\mathcal{L}}$ describing magnetization transfer between an aqueous pool $l$ and its associated non-aqueous pool $m$,
$\boldsymbol{\Lambda}_0^{l,m}, \boldsymbol{\Lambda}^{l,m}$: submatrices of $\boldsymbol{\mathcal{L}}$ representing the BSB model with a water pool $l$ and a macromolecular pool $m$,
$\mu_0$: magnetic permeability of free space,
$\boldsymbol{\mu}_0$: vector specifying the mean direction of the fiber orientation distribution,
$\boldsymbol{\mu}_{1,2}$: vectors characterizing the width and ovality of the Bingham distribution,
$\Delta\nu$: offset frequency in Hz,
$\tau_{1,2}^{-1}$: $T_1$ and $T_2$ relaxographic shutter speeds,
$\tau_c$: correlation time,
$\tau_c^\infty$: pre-exponential factor,
$\tau_f$: inter-pulse delay in the GS module,
$\tau_m$: mixing time for the MW and IEW pools,
$\tau_p$: RF pulse length,
$\tau_s$: inter-pulse separation in the MT pulse train,
$\phi$: azimuth angle,
$\phi_A$: azimuth angle in the fiber frame,
$\Omega$: offset frequency (in rad/s),
$\omega_0$: Larmor frequency,
$\boldsymbol{\omega}_1$: RF field vector expressed in rad/s,
$\omega_1$: RF field amplitude expressed in rad/s,
$\omega_{1x}, \omega_{1y}$: Cartesian coordinates of the RF field amplitude expressed in rad/s,
$\omega_{\mathrm{RF}}$: frequency of the RF field (in rad/s),
$(\ldots)^T$: transpose of a matrix.

# Supplementary Methods

## Dynamic Matrix for the Four-Pool Model

The entire 12×12 dynamic matrix for the 4PM is given by

$$\boldsymbol{\mathcal{L}} = \boldsymbol{\ell} + \boldsymbol{k}, \tag{S1}$$

where

$$\boldsymbol{\ell} = \begin{pmatrix} 0 & 0 & 0 & 0 & 0 & 0 & 0 & 0 & 0 & 0 & 0 & 0 \\ 0 & R_2^{\mathrm{IEW}} & \Omega & -\omega_{1y} & 0 & 0 & 0 & 0 & 0 & 0 & 0 & 0 \\ 0 & -\Omega & R_2^{\mathrm{IEW}} & \omega_{1x} & 0 & 0 & 0 & 0 & 0 & 0 & 0 & 0 \\ -2R_1^{\mathrm{IEW}}M_0^{\mathrm{IEW}} & \omega_{1y} & -\omega_{1x} & R_1^{\mathrm{IEW}} & 0 & 0 & 0 & 0 & 0 & 0 & 0 & 0 \\ -2R_1^{\mathrm{NM}}M_0^{\mathrm{NM}} & 0 & 0 & 0 & R_1^{\mathrm{NM}} + R_{\mathrm{RF}}^{\mathrm{NM}} & 0 & 0 & 0 & 0 & 0 & 0 & 0 \\ 0 & 0 & 0 & 0 & 0 & R_2^{\mathrm{MW}} & \Omega & -\omega_{1y} & 0 & 0 & 0 & 0 \\ 0 & 0 & 0 & 0 & 0 & -\Omega & R_2^{\mathrm{MW}} & \omega_{1x} & 0 & 0 & 0 & 0 \\ -2R_1^{\mathrm{MW}}M_0^{\mathrm{MW}} & 0 & 0 & 0 & 0 & \omega_{1y} & -\omega_{1x} & R_1^{\mathrm{MW}} & 0 & 0 & 0 & 0 \\ -2R_1^{\mathrm{M}}M_0^{\mathrm{M}} & 0 & 0 & 0 & 0 & 0 & 0 & 0 & R_1^{\mathrm{M}} + R_{\mathrm{RF}}^{\mathrm{M}} & 0 & 0 & 0 \\ 0 & 0 & 0 & 0 & 0 & 0 & 0 & 0 & 0 & R_2^{\mathrm{BW}} & \Omega & -\omega_{1y} \\ 0 & 0 & 0 & 0 & 0 & 0 & 0 & 0 & 0 & -\Omega & R_2^{\mathrm{BW}} & \omega_{1x} \\ -2R_1^{\mathrm{BW}}M_0^{\mathrm{BW}} & 0 & 0 & 0 & 0 & 0 & 0 & 0 & 0 & \omega_{1y} & -\omega_{1x} & R_1^{\mathrm{BW}} \end{pmatrix} \tag{S2}$$

describes the intrinsic relaxation processes (in the absence of exchange) of all pools $l \in \{\mathrm{IEW, NM, MW, M, BW}\}$ and the effects from RF pulses, and

$$\boldsymbol{k} = \begin{pmatrix} 0 & 0 & 0 & 0 & 0 & 0 & 0 & 0 & 0 & 0 & 0 & 0 \\ 0 & k_{\mathrm{IEW,MW}} + k_{\mathrm{IEW,NM}} & 0 & 0 & 0 & -k_{\mathrm{MW,IEW}} & 0 & 0 & 0 & 0 & 0 & 0 \\ 0 & 0 & k_{\mathrm{IEW,MW}} + k_{\mathrm{IEW,NM}} & 0 & 0 & 0 & -k_{\mathrm{MW,IEW}} & 0 & 0 & 0 & 0 & 0 \\ 0 & 0 & 0 & k_{\mathrm{IEW,MW}} + k_{\mathrm{IEW,NM}} & -k_{\mathrm{NM,IEW}} & 0 & 0 & -k_{\mathrm{MW,IEW}} & 0 & 0 & 0 & 0 \\ 0 & 0 & 0 & -k_{\mathrm{IEW,NM}} & k_{\mathrm{NM,IEW}} & 0 & 0 & 0 & 0 & 0 & 0 & 0 \\ 0 & -k_{\mathrm{IEW,MW}} & 0 & 0 & 0 & k_{\mathrm{MW,IEW}} + k_{\mathrm{MW,M}} & 0 & 0 & 0 & 0 & 0 & 0 \\ 0 & 0 & -k_{\mathrm{IEW,MW}} & 0 & 0 & 0 & k_{\mathrm{MW,IEW}} + k_{\mathrm{MW,M}} & 0 & 0 & 0 & 0 & 0 \\ 0 & 0 & 0 & -k_{\mathrm{IEW,MW}} & 0 & 0 & 0 & k_{\mathrm{MW,IEW}} + k_{\mathrm{MW,M}} & -k_{\mathrm{M,MW}} & 0 & 0 & 0 \\ 0 & 0 & 0 & 0 & 0 & 0 & 0 & -k_{\mathrm{MW,M}} & k_{\mathrm{M,MW}} & 0 & 0 & 0 \\ 0 & 0 & 0 & 0 & 0 & 0 & 0 & 0 & 0 & 0 & 0 & 0 \\ 0 & 0 & 0 & 0 & 0 & 0 & 0 & 0 & 0 & 0 & 0 & 0 \\ 0 & 0 & 0 & 0 & 0 & 0 & 0 & 0 & 0 & 0 & 0 & 0 \end{pmatrix} \tag{S3}$$

describes all exchange processes.

For an expanded 2PM consisting of an aqueous pool $A$ and a non-aqueous semisolid pool $B$ as well as an additional bulk water pool BW, $\boldsymbol{\mathcal{L}}$ is a square matrix of order 8 with

$$\boldsymbol{\mathcal{L}} = \boldsymbol{\ell} + \boldsymbol{k} = \begin{pmatrix} 0 & 0 & 0 & 0 & 0 & 0 & 0 & 0 \\ 0 & R_2^A + k_{A,B} & \Omega & -\omega_{1y} & 0 & 0 & 0 & 0 \\ 0 & -\Omega & R_2^A + k_{A,B} & \omega_{1x} & 0 & 0 & 0 & 0 \\ -2R_1^A M_0^A & \omega_{1y} & -\omega_{1x} & R_1^A + k_{A,B} & -k_{B,A} & 0 & 0 & 0 \\ -2R_1^B M_0^B & 0 & 0 & -k_{A,B} & R_1^B + k_{B,A} + R_{\mathrm{RF}}^B & 0 & 0 & 0 \\ 0 & 0 & 0 & 0 & 0 & R_2^{\mathrm{BW}} & \Omega & -\omega_{1y} \\ 0 & 0 & 0 & 0 & 0 & -\Omega & R_2^{\mathrm{BW}} & \omega_{1x} \\ -2R_1^{\mathrm{BW}} M_0^{\mathrm{BW}} & 0 & 0 & 0 & 0 & \omega_{1y} & -\omega_{1x} & R_1^{\mathrm{BW}} \end{pmatrix}. \tag{S4}$$

## Absorption lineshapes of the semisolid proton pools

In the current work, the effect of RF pulses on the non-aqueous pools $m \in \{\text{NM}, \text{M}\}$ was expressed by an RF absorption parameter $R_{\text{RF}}^{m} = \pi\omega_1^2 g^m(\Omega, T_2^m)$, and was modeled using a super-Lorentzian lineshape (43):

$$g_{sL}^{m}(\Omega, T_2^m) = \frac{1}{\sqrt{\pi}} \int_0^{\pi/2} \frac{\sqrt{2}T_2^m}{|3\cos^2\theta - 1|} \exp\left[-\left(\frac{\sqrt{2}T_2^m\Omega}{|3\cos^2\theta - 1|}\right)^2\right] \sin\theta \, d\theta. \quad \text{(S5)}$$

Alternatively, for the non-aqueous myelin pool M, a "Bingham lineshape" (25),

$$g_B^{\text{M}}(\Omega, T_2^{\text{M}}) = \mathcal{N} \int_0^{\pi} \int_0^{2\pi} g_{\text{cyl}}^{\text{M}}(\Omega, \theta, T_2^{\text{M}}) \sum_i \beta_i(\theta, \phi) \sin\theta \, d\theta d\phi, \quad \text{(S6)}$$

was also used. $\mathcal{N}$ is a normalization factor that ensures $\int g_B^{\text{M}}(\Omega, T_2^{\text{M}}) d\Omega = 1$ and $i \geq 1$ denotes the number of fODF peaks assumed to reflect different fiber bundles inside the voxel. The lineshape of a single cylindrical fiber is given by

$$g_{\text{cyl}}^{\text{M}}(\Omega, \theta, T_2^{\text{M}}) = \frac{1}{(2\pi)^{3/2}} \int_0^{2\pi} \frac{1}{\sqrt{(R_2^{\text{res}})^2 + (r_2^{\text{M}})^2}} \exp\left[-\frac{1}{2} \cdot \frac{\Omega^2}{(R_2^{\text{res}})^2 + (r_2^{\text{M}})^2}\right] d\phi_A, \quad \text{(S7)}$$

where $\phi_A$ is the azimuth of the membrane normal in a "fiber frame", whose $z$-axis is defined by the cylinder axis. The width of the "fiber lineshape" is characterized by

$$r_2^{\text{M}}(\theta, \phi_A) = \frac{|3\cos^2\phi_A \sin^2\theta - 1|}{2T_2^{\text{M}}} \quad \text{(S8)}$$

and a rate constant $R_2^{\text{res}}$ that corresponds to the residual width of a bilayer segment oriented at the magic angle.

## Supplementary Tables

**Supplementary Table S1. Acquisition parameters used in the relaxometry experiments.** A full description of the abbreviations of sequence building blocks and parameters is given in the Methods section.

| # | Sequence type | MT pulses (preparation module) | Preparation pulse | MT pulses (evolution module) |
|---|---|---|---|---|
| 1 | IR | – | RECT, $\tau_p$ = 40 µs | – |
| 2 | IR | – | BIR-4, $\tau_p$ = 5 ms | – |
| 3 | Transient MT | – | MT-sat, $\tau_p$ = 6 ms | – |
| 4 | Steady-state MT ($N_{\text{RF}}$ = 0, 20, …, 300) | $\gamma B_{1,\text{RMS}}/(2\pi)$ = 500 Hz<br>$\tau_s$ = 250 µs | – | – |
| 5 | | $\gamma B_{1,\text{RMS}}/(2\pi)$ = 500 Hz<br>$\tau_s$ = 2250 µs | | – |
| 6 | | $\gamma B_{1,\text{RMS}}/(2\pi)$ = 500 Hz<br>$\tau_s$ = 4250 µs | | – |
| 7 | Steady-state MT ($N_{\text{RF}}$ = 0, 20, …, 300) | $\gamma B_{1,\text{RMS}}/(2\pi)$ = 750 Hz<br>$\tau_s$ = 250 µs | – | – |
| 8 | | $\gamma B_{1,\text{RMS}}/(2\pi)$ = 750 Hz<br>$\tau_s$ = 2250 µs | | – |
| 9 | | $\gamma B_{1,\text{RMS}}/(2\pi)$ = 750 Hz<br>$\tau_s$ = 4250 µs | | – |
| 10 | Steady-state MT ($N_{\text{RF}}$ = 0, 20, …, 300) | $\gamma B_{1,\text{RMS}}/(2\pi)$ = 1000 Hz<br>$\tau_s$ = 250 µs | – | – |
| 11 | | $\gamma B_{1,\text{RMS}}/(2\pi)$ = 1000 Hz<br>$\tau_s$ = 2250 µs | | – |
| 12 | | $\gamma B_{1,\text{RMS}}/(2\pi)$ = 1000 Hz<br>$\tau_s$ = 4250 µs | | – |
| 13 | GS ‘up’ | – | $\tau_f$ = 1 ms | – |
| 14 | GS ‘down’ | – | $\tau_f$ = 1 ms | – |
| 15 | GS ‘up’ | – | $\tau_f$ = 50 ms | – |
| 16 | GS ‘down’ | – | $\tau_f$ = 50 ms | – |
| 17 | MT-prepared IR ($N_{\text{RF}}$ = 250) | $\gamma B_{1,\text{RMS}}/(2\pi)$ = 500 Hz<br>$\tau_s$ = 250 µs | RECT, $\tau_p$ = 40 µs | $\gamma B_{1,\text{RMS}}/(2\pi)$ = 500 Hz<br>$\tau_s$ = 250 µs |
| 18 | | | – | |
| 19 | MT-prepared IR ($N_{\text{RF}}$ = 250) | $\gamma B_{1,\text{RMS}}/(2\pi)$ = 500 Hz<br>$\tau_s$ = 2250 µs | RECT, $\tau_p$ = 40 µs | $\gamma B_{1,\text{RMS}}/(2\pi)$ = 500 Hz<br>$\tau_s$ = 2250 µs |
| 20 | | | – | |
| 21 | MT-prepared IR ($N_{\text{RF}}$ = 250) | $\gamma B_{1,\text{RMS}}/(2\pi)$ = 500 Hz<br>$\tau_s$ = 4250 µs | RECT, $\tau_p$ = 40 µs | $\gamma B_{1,\text{RMS}}/(2\pi)$ = 500 Hz<br>$\tau_s$ = 4250 µs |
| 22 | | | – | |

**Supplementary Table S2. Results from voxel-wise fitting Bingham parameters to the diffusion-weighted data.** Values were averaged over the sample (excluding edges). The orientation $\theta_0$ is derived from the mean direction of the largest component. The analysis resulted in three components, where the two smaller are safely considered as artifacts of the fitting procedure. It must remain unclear, weather the temperature dependence of the parameters is really real or whether it is an artifact. With this in mind and considering potential uncertainty in the fit, it was assumed that the fODF can be approximated by a cylindrically symmetric distribution with one component and setting $\kappa_1 \approx \kappa_2 \approx 5$. This value is used for all computations considering a Bingham distribution in the entire study.

| **Temperature** | **Bingham parameters** (sample averaged±standard deviation) | | |
|---|---|---|---|
| | $\kappa_1$ | $\kappa_2$ | $\theta_0/°$ |
| 21°C | 4.4±0.1 | 4.6±0.1 | 72.9±0.8 |
| 28°C | 4.8±0.2 | 5.2±0.1 | 73±0.9 |
| 35°C | 5.2±0.2 | 5.6±0.1 | 73±0.9 |

**Supplementary Table S3. Results from fitting the propagator matrix elements $\mathcal{P}_{5,5}^{(i)}$ and $\mathcal{P}_{9,9}^{(i)}$.** They act on the non-aqueous pools $m \in \{\mathrm{NM}, \mathrm{M}\}$ (see Eqs. 11 and 12) for all RF pulse events with $\Delta\nu$=0 (i.e., on resonance). The values are expressed as effective flip angles $\alpha_{\mathrm{eff}}^{m}$ in degrees. If the same RF pulse was used in multiple sequences, such as the initial 20 µs rectangular readout pulse (nominal 90°) and the composite refocusing pulses (nominal 180°) of the CPMG train, the same effective flip angle was assumed for all sequences. Note that no potential temperature dependence was considered for the CPMG refocusing pulses because the results were largely insensitive to this parameter. In general, the estimations obtained for the two non-aqueous pools were highly correlated for each pulse type. Thus, the differences between $\alpha_{\mathrm{eff}}^{\mathrm{nM}}$ and $\alpha_{\mathrm{eff}}^{\mathrm{M}}$ are likely to be subject to further systematic errors.

| **Temperature-independent parameters** | | | |
|---|---|---|---|
| Composite CPMG refocusing pulse | | | |
| $\alpha_{\mathrm{eff}}^{\mathrm{NM}}$ / ° | 178.3 ± 3.7 | | |
| $\alpha_{\mathrm{eff}}^{\mathrm{M}}$ / ° | 180 ± 2.4 | | |
| **Temperature-dependent parameters** | | | |
| | **21 °C** | **28 °C** | **35 °C** |
| Initial 20µs CPMG rectangular readout pulse | | | |
| $\alpha_{\mathrm{eff}}^{\mathrm{NM}}$ / ° | 81.0 ± 4.2 | 74.8 ± 2.9 | 75.3 ± 3.2 |
| $\alpha_{\mathrm{eff}}^{\mathrm{M}}$ / ° | 74.7 ± 2.8 | 80.5 ± 3.5 | 78.0 ± 3.0 |
| 40µs rectangular inversion pulse (“RECT”) | | | |
| $\alpha_{\mathrm{eff}}^{\mathrm{NM}}$ / ° | 180 ± 2.2 | 161.9 ± 2.4 | 179.9 ± 2.6 |
| $\alpha_{\mathrm{eff}}^{\mathrm{M}}$ / ° | 122.0 ± 1.3 | 139.9 ± 1.4 | 137.5 ± 1.5 |
| 5ms adiabatic inversion pulse (“BIR-4”) | | | |
| $\alpha_{\mathrm{eff}}^{\mathrm{NM}}$ / ° | 108.2 ± 2.1 | 107.6 ± 2.3 | 111.7 ± 2.5 |
| $\alpha_{\mathrm{eff}}^{\mathrm{M}}$ / ° | 63.6 ± 1.3 | 69.3 ± 1.5 | 67.2 ± 1.5 |
| Composite saturation pulse for transient MT experiments | | | |
| $\alpha_{\mathrm{eff}}^{\mathrm{NM}}$ / ° | 86.0 ± 2.1 | 83.2 ± 2.3 | 82.5 ± 2.5 |
| $\alpha_{\mathrm{eff}}^{\mathrm{M}}$ / ° | 92.9 ± 1.3 | 92.9 ± 1.4 | 92.9 ± 1.5 |

**Supplementary Table S4. Summary of the fitted parameters for a 2PM model.** It consists of an aqueous proton pool $A$ and a nonaqueous semisolid pool $B$. As for the 4PM, the results were obtained by combined analysis of the experiments performed at three temperatures with a total of 24 free parameters. The pool-size fractions were considered to be temperature-independent. Since only $\Delta\nu = \pm 15$ kHz was utilized in the acquisition of relaxometry data (Table 1; protocols 17–22), the fits did not require the assumption of a specific absorption lineshape for the nonaqueous pool. For easier comparisons to literature values, $T_2^B$ corresponds to a super-Lorentzian, Eq. S5, which is typically assumed for WM.

| **Temperature-independent parameters** | | | |
|---|---|---|---|
| Fractional pool sizes $A^l$ | | Corrected fractional pool sizes $A_c^l$ in WM | |
| $A^A$ | 0.739 ± 0.004* | $A_c^A$ | 0.764 ± 0.004 |
| $A^B$ | 0.228 ± 0.004 | $A_c^B$ | 0.236 ± 0.004 |
| $A^{BW}$ | 0.033 ± 0.001 | | — |

| **Temperature-dependent parameters** | **21 °C** | **28 °C** | **35 °C** |
|---|---|---|---|
| Exchange rate $k_{l\leftrightarrow m}$ | | | |
| $k_{A\leftrightarrow B}$ / s$^{-1}$ | 16.59 ± 0.22 | 16.61 ± 0.22 | 17.28 ± 0.23 |
| Longitudinal relaxation rates $R_1^l$ | | | |
| $R_1^A$ / s$^{-1}$ | 0.94 ± 0.02 | 0.87 ± 0.02 | 0.81 ± 0.02 |
| $R_1^B$ / s$^{-1}$ | 2.28 ± 0.07 | 2.58 ± 0.07 | 2.64 ± 0.07 |
| $R_1^{BW}$ / s$^{-1}$ | *(0.44 ± 0.01)*† | *(0.44 ± 0.01)*† | *(0.44 ± 0.01)*† |
| Transverse relaxation rates $R_2^l$ (aqueous pools) | | | |
| $R_2^A$ / s$^{-1}$ | 13.38 ± 0.04 | 14.14 ± 0.04 | 15.30 ± 0.05 |
| $R_2^{BW}$ / s$^{-1}$ | 0.98 ± 0.08 | 1.67 ± 0.08 | *(2.00 ± 0.08)*† |
| Transverse relaxation time $T_2^l$ (nonaqueous pool) | | | |
| $T_2^B$ / µs | 14.13 | 14.05 | 14.17 |

* The confidence interval was not obtained directly from the fit but estimated by error propagation.

† The estimated relaxation time of the BW pool assumed a boundary value.

**Supplementary Table S5. Summary of activation energies.** Estimates were obtained from the temperature-dependent variation of the 4PM exchange rates and relaxation constants assuming Arrhenius behavior. Uncertainties in the activation energies represent the 68% confidence interval.

| **Parameter** | **$E_a$ [kJ/mol]** | **$r^2$** | **Assumed process** |
|---|---|---|---|
| $k_{\mathrm{IEW}\leftrightarrow\mathrm{MW}}$ | 29.4±5.0 | **0.992** | Transmembrane diffusion of water |
| $k_{\mathrm{IEW}\leftrightarrow\mathrm{NM}}$ | 6.8±2.9 | **0.953** | Proton exchange between water and lipid head groups |
| $k_{\mathrm{MW}\leftrightarrow\mathrm{M}}$ | *Unreliable* | *0.405* | |
| $R_1^{\mathrm{IEW}}$ | 10.4±0.8 | **0.998** | Reorientation of water |
| $R_1^{\mathrm{MW}}$ | 6.0±3.7 | 0.902 | Translational motion of clusters of hydration water |
| $R_1^{\mathrm{NM}}$ | *Unreliable* | *0.805* | |
| $R_1^{\mathrm{M}}$ | *Unreliable* | *0.109* | |
| $R_2^{\mathrm{IEW}}$ | –8.0±0.5 | **0.999** | Proton exchange between water and lipid head groups |
| $R_2^{\mathrm{MW}}$ | –6.2±1.9 | **0.975** | Proton exchange between water and lipid head groups |

**Supplementary Table S6. Subset of 4PM parameters obtained from additional analyses with fits of $T_2^{\mathrm{nM}}$, $T_2^{\mathrm{M}}$ and $k_{\mathrm{MW}\leftrightarrow\mathrm{M}}$ to *z*-spectra.** The *z*-spectra were acquired with cosine-modulated MT pulses; all other 4PM parameters were fixed (Table 2). The results are compared to forward simulations without such adjustments (Approach 1). Approaches 2 and 3 differ in the assumed absorption lineshapes $g^m(\Omega, T_2^m)$ for the two nonaqueous pools, which were either super-Lorentzian lineshapes, $g_{sL}^{\mathrm{NM}}(T_2^{\mathrm{NM}})$ and $g_{sL}^{\mathrm{M}}(T_2^{\mathrm{M}})$, for both pools (Approach 2), or a super-Lorentzian $g_{sL}^{\mathrm{NM}}(T_2^{\mathrm{NM}})$ and a Bingham lineshape $g_B^{\mathrm{M}}(T_2^{\mathrm{M}})$ for the NM and M pool, respectively (Approach 3). Note that due to the strong correlation between the fitted rate constants $k_{\mathrm{MW}\leftrightarrow\mathrm{W}}$ and $k_{\mathrm{IEW}\leftrightarrow\mathrm{NM}}$, further refinement by readjusting also $k_{\mathrm{IEW}\leftrightarrow\mathrm{NM}}$ does not yield robust improvement and was, therefore, omitted.

| | **Approach 1** $g_{sL}^{\mathrm{nM}}(T_2^{\mathrm{NM}})$ \| $g_{sL}^{\mathrm{M}}(T_2^{\mathrm{M}})$ | **Approach 2** $g_{sL}^{\mathrm{NM}}(T_2^{\mathrm{NM}})$ \| $g_{sL}^{\mathrm{M}}(T_2^{\mathrm{M}})$ | **Approach 3** $g_{sL}^{\mathrm{nM}}(T_2^{\mathrm{nM}})$ \| $g_B^{\mathrm{M}}(T_2^{\mathrm{M}})$ |
|---|---|---|---|
| | | $T$ = 21 °C | |
| $T_2^{\mathrm{NM}}$ / µs | 13.0 | 15.4 | 15.8 |
| $T_2^{\mathrm{M}}$ / µs | 13.3 | 10.2 | 11.0 |
| $k_{\mathrm{MW}\leftrightarrow\mathrm{M}}$ / s$^{-1}$ | 36.8 | 49.0 | 50.7 |
| | | $T$ = 28 °C | |
| $T_2^{\mathrm{NM}}$ / µs | 12.7 | 14.6 | 15.2 |
| $T_2^{\mathrm{M}}$ / µs | 13.8 | 11.4 | 12.1 |
| $k_{\mathrm{MW}\leftrightarrow\mathrm{M}}$ / s$^{-1}$ | 32.9 | 32.3 | 33.2 |
| | | $T$ = 35 °C | |
| $T_2^{\mathrm{NM}}$ / µs | 12.7 | 16.7 | 17.2 |
| $T_2^{\mathrm{M}}$ / µs | 14.3 | 11.3 | 12.1 |
| $k_{\mathrm{MW}\leftrightarrow\mathrm{M}}$ / s$^{-1}$ | 34.3 | 48.6 | 50.2 |

**Supplementary Table S7. Starting values as well as lower and upper bounds considered for the four-pool-model fit.** The same parameters were used for all temperatures. The bounds did not constrain the estimated parameters for the tissue. However, some of the fitted parameters for the additional BW pool tended to fall on one of the bounds.

| Parameter | Starting value | Lower bound | Upper bound |
|---|---|---|---|
| Fractional pool sizes $A^l$ | | | |
| $A^{\mathrm{IEW}}$ | 0.55 | 0 | 1 |
| $A^{\mathrm{MW}}$ | 0.50 | 0 | 1 |
| $A^{\mathrm{nM}}$ | 0.25 | 0 | 1 |
| $A^{\mathrm{M}}$ | 0.25 | 0 | 1 |
| $A^{\mathrm{BW}}$ | 0.05 | 0 | 1 |
| Exchange rates $k_{l\leftrightarrow m}$ | | | |
| $k_{\mathrm{IEW}\leftrightarrow\mathrm{MW}}$ / $s^{-1}$ | 5 | 0 | 70 |
| $k_{\mathrm{IEW}\leftrightarrow\mathrm{nM}}$ / $s^{-1}$ | 5 | 0 | 70 |
| $k_{\mathrm{MW}\leftrightarrow\mathrm{M}}$ / $s^{-1}$ | 5 | 0 | 70 |
| Longitudinal relaxation rates $R_1^l$ | | | |
| $R_1^{\mathrm{IEW}}$ / $s^{-1}$ | 1 | 0.4 | 2 |
| $R_1^{\mathrm{MW}}$ / $s^{-1}$ | 1 | 0.4 | 10 |
| $R_1^{\mathrm{nM}}$ / $s^{-1}$ | 3 | 1 | 5 |
| $R_1^{\mathrm{M}}$ / $s^{-1}$ | 3 | 1 | 5 |
| $R_1^{\mathrm{BW}}$ / $s^{-1}$ | 0.25 | 0.166 | 0.444 |
| Transverse relaxation rates $R_2^l$ (aqueous pools) | | | |
| $R_2^{\mathrm{IEW}}$ / $s^{-1}$ | 20 | 4 | 25 |
| $R_2^{\mathrm{MW}}$ / $s^{-1}$ | 100 | 20 | 500 |
| $R_2^{\mathrm{BW}}$ / $s^{-1}$ | 2 | 0.5 | 2 |
| Transverse relaxation times $T_2^l$ (non-aqueous pools) | | | |
| $T_2^{\mathrm{nM}}$ / µs | 10 | 1 | 30 |
| $T_2^{\mathrm{M}}$ / µs | 10 | 1 | 30 |

## Supplementary Figures

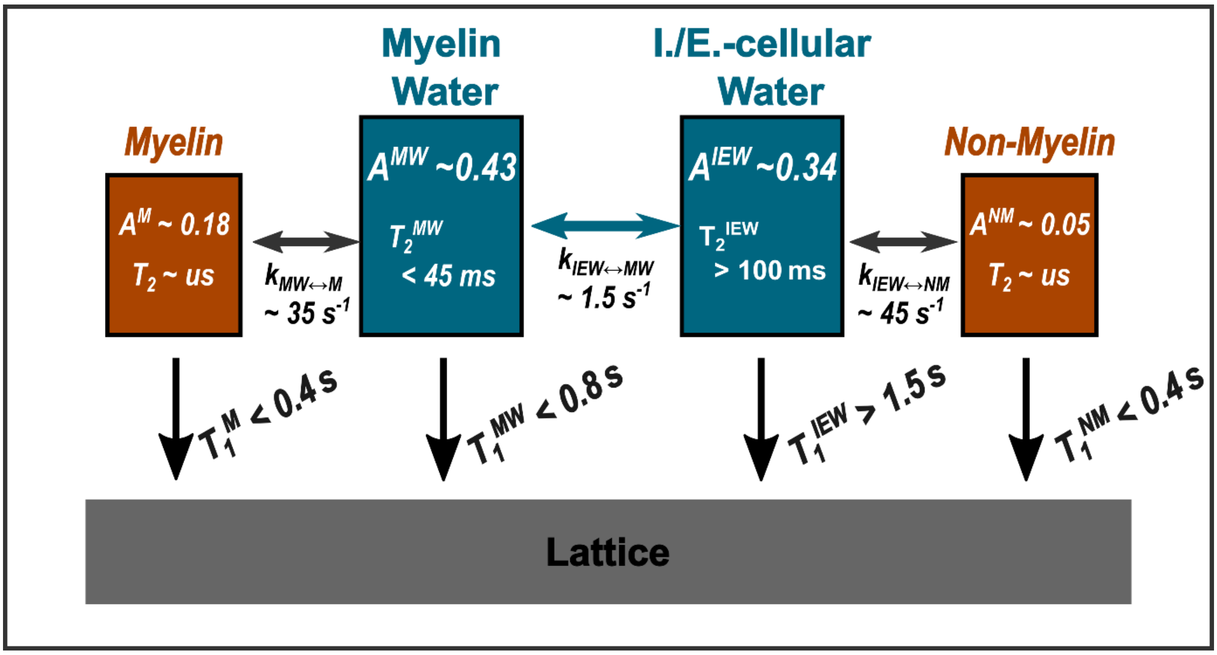


**Supplementary Figure S1. Schematic of the 4PM of WM.** It considers two aqueous proton pools, namely "myelin water" (MW) and "intra-/extracellular water" (IEW), and associated nonaqueous pools, referred to as "nonaqueous myelin" pool (M) and "nonaqueous non-myelin" pool (NM). The indicated parameter estimates of the fractional pool sizes, $A^l$ ($l \in$ {IEW,NM,MW,M}), relaxation times, $T_1^l$ and $T_2^l$, and exchange rates, $k_{l\leftrightarrow m}$, correspond to results of the current measurements in fixed porcine spinal cord.

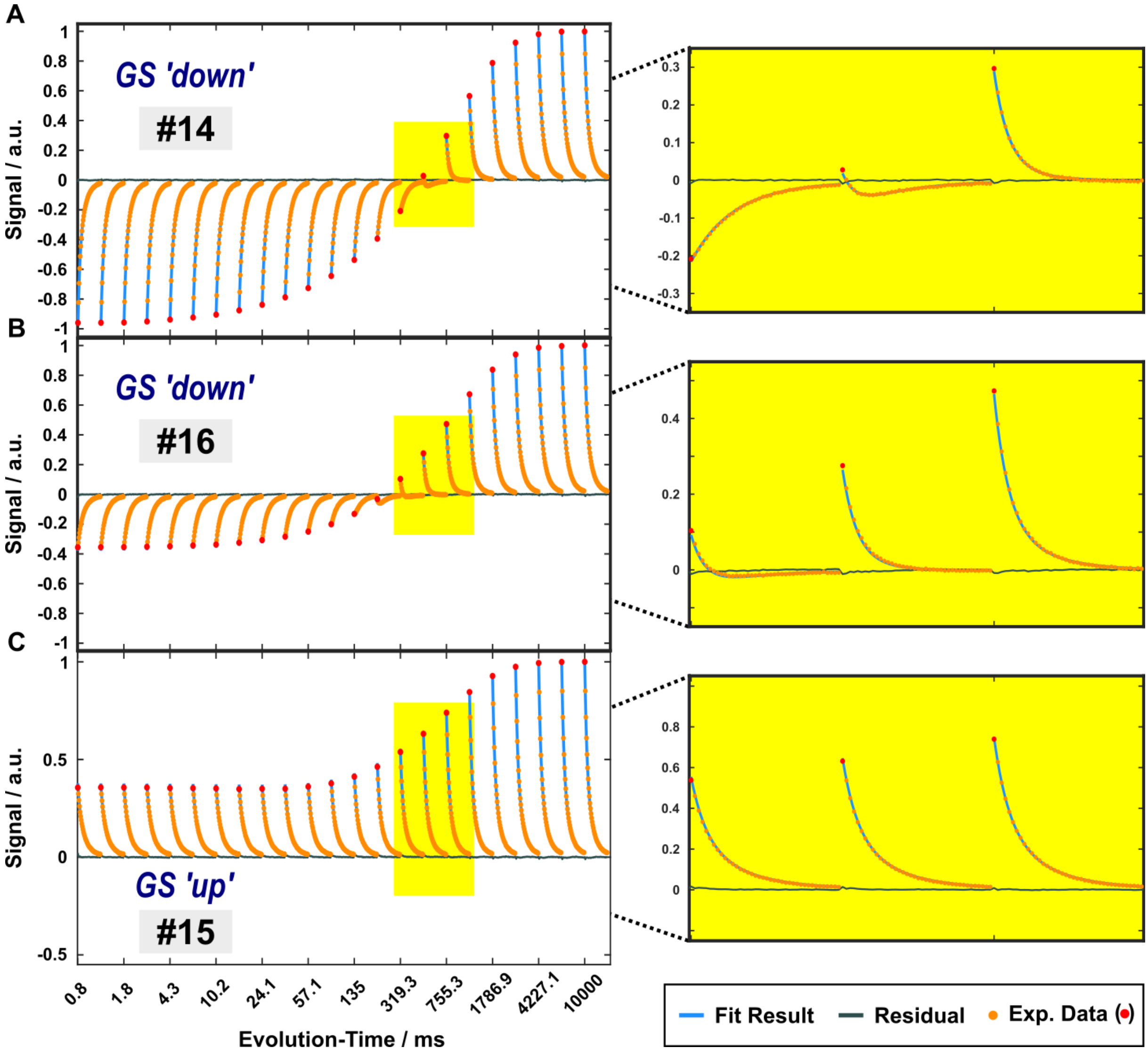


**Supplementary Figure S2. Examples of Goldman-Shen filtering experiments.** Data were recorded at 35 °C with GS 'down' and filter times of 1 ms **(A)** and 50 ms **(B)** as well as GS 'up' with the longer filter time of 50 ms (**C**). The experimental signals of the first echoes are shown as red circles and the remaining CPMG train as orange circles (only every second data point of the densely acquired echoes is plotted for better visibility). Corresponding fits (blue solid lines) and residuals (gray solid lines) are displayed as in Figure 4. The right column shows a zoomed region corresponding to the region indicated by yellow shading.

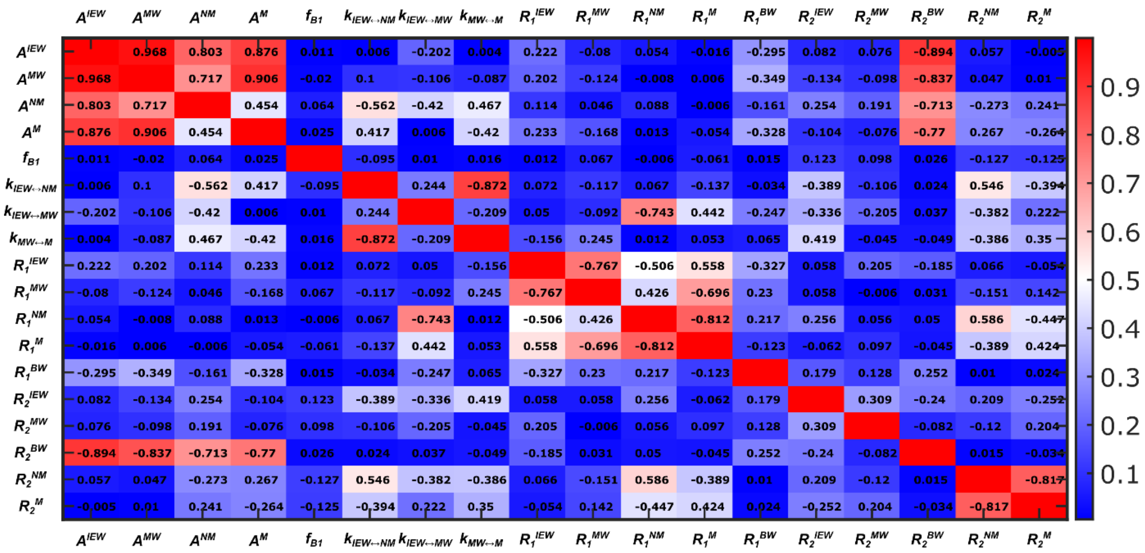


**Supplementary Figure S3. Color-coded correlation matrix of a subset of 4PM parameters.** The parameters (results for 35 °C) were obtained from simultaneous fitting of the concatenated data from all 22 protocols (Table 1). As expected, the highest correlations were found between relative pool sizes (due to the normalization).

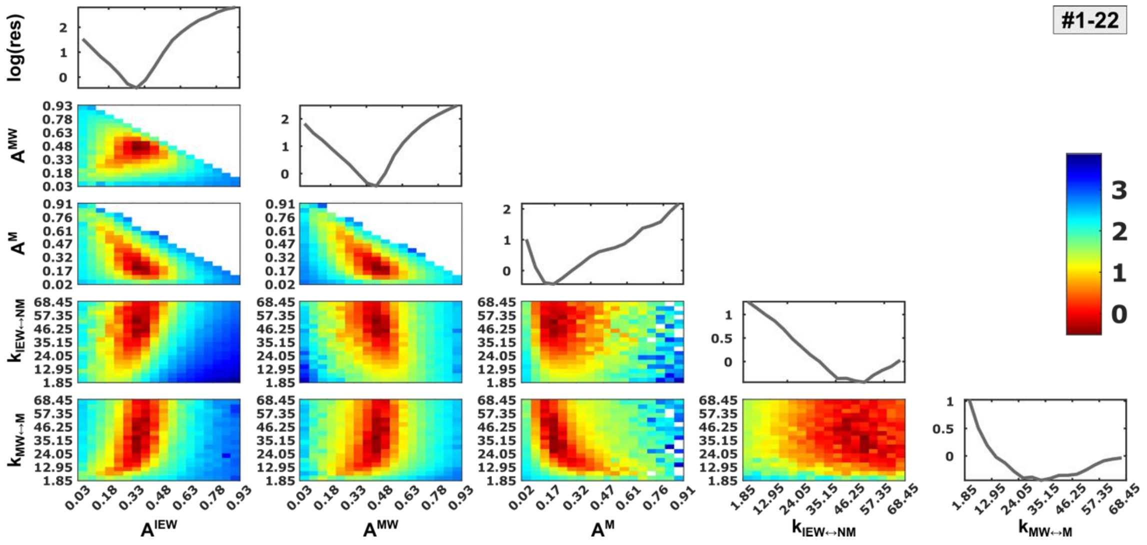


**Supplementary Figure S4. 'Landscape plots' of residuals of the 4PM parameters with the highest nominal pairwise correlations.** The residuals (color-coded in log scale from –0.51 to 3.87) refer to the data acquired at 35 °C (see Supplementary Figure S2). In particular, 32,768 four-pool models were simulated with random variation of the fractional pool sizes $A^{\mathrm{IEW}}$ (between $5\times10^{-5}$ and 0.941), $A^{\mathrm{MW}}$ (between $2\times10^{-}$ and 0. 945) and $A^{\mathrm{M}}$ (between $1\times10^{-5}$ and 0.922) as well as exchange rates $k_{\mathrm{IEW}\leftrightarrow\mathrm{NM}}$ (between 0.009 and 70 $s^{-1}$) and $k_{\mathrm{MW}\leftrightarrow\mathrm{M}}$ (between 0.008 and 70.0 $s^{-1}$), and the squared L2-norm was calculated. A rather flat hypersurface (e.g., for $k_{\mathrm{MW}\leftrightarrow\mathrm{M}}$ vs. $k_{\mathrm{IEW}\leftrightarrow\mathrm{NM}}$) indicates a relevant risk of bias due to parameter correlation whereas a deep minimum (e.g., for $A^{\mathrm{IEW}}$ vs. $A^{\mathrm{MW}}$ or $A^{\mathrm{M}}$ vs. $A^{\mathrm{MW}}$) suggests higher accuracy. White areas represent non-meaningful parameter combinations.

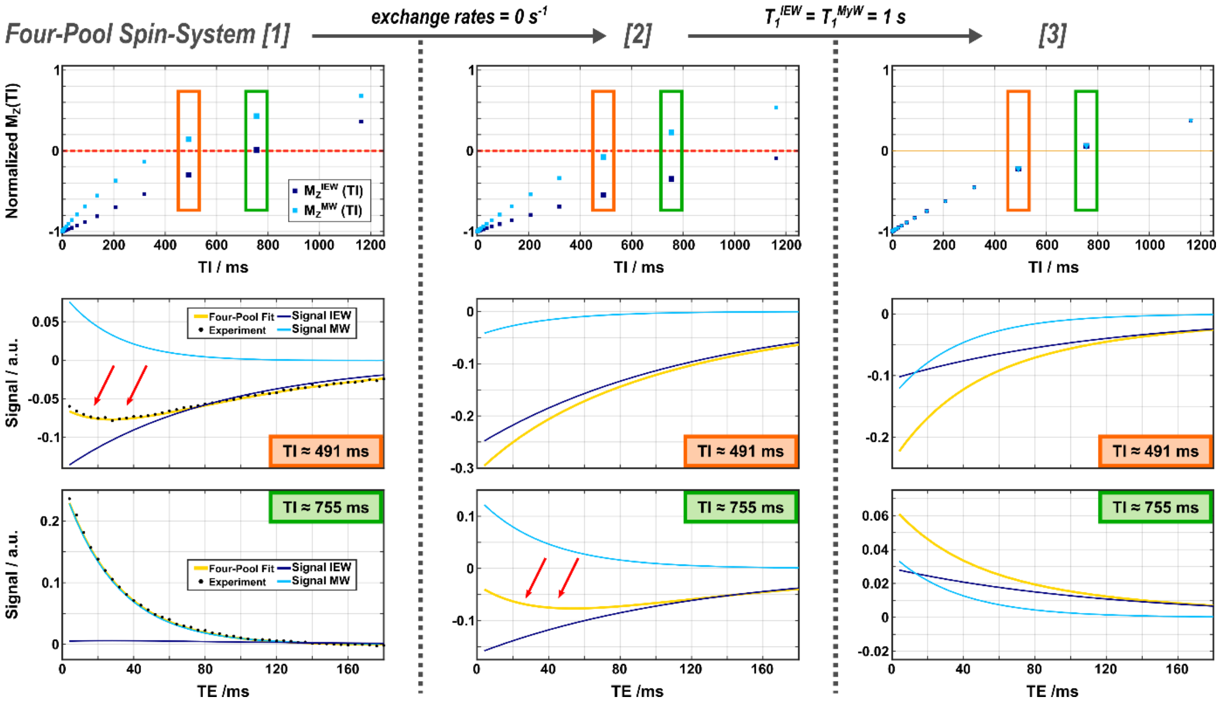


**Supplementary Figure S5. Simulations of transient decays of the echo amplitude in IR-prepared CPMG experiments.** The **top row** shows the computed recovery of the longitudinal magnetizations for both water pools (individually normalized to 1 for TI → ∞) based on the fits (Table 2) to the data measured at 35 °C with protocol 1 (IR-prepared CPMG echo train, hard inversion pulse). Further shown are TE-dependent transients of the echo amplitudes at TI=491 ms, near the zero-crossing of the experimental recovery curve **(middle row)**, and at TI=755 ms **(bottom row)**. The experimental echo transients, corresponding to the combined contributions from both water pools (shown as black dots) are perfectly reproduced by the 4PM prediction **(left column)**. For TI=491 ms, the echo amplitude passes through a minimum (red arrows) due to the superposition of a positive contribution from $M_z^{\mathrm{MW}}$ and a negative contribution from $M_z^{\mathrm{IEW}}$, which both relax towards zero with increasing TE **(left column)**. In the absence of exchange (i.e., all $k_{l,m}$ set to zero) longitudinal relaxation of the water pools after the inversion pulse would be slower, shifting the zero-crossing region to a longer TI around 755 ms, where a similar transient minimum of the echo amplitude (red arrows) would be observed (**middle column**). Without exchange and for equal longitudinal relaxation times of the water pools (here, $T_1^{\mathrm{IEW}} = T_1^{\mathrm{MW}} = 1$ s), the relaxation of $M_z^{\mathrm{MW}}$ and $M_z^{\mathrm{IEW}}$ would be synchronized with identical zero-crossings. Hence, their contributions would always have the same sign (i.e., signal phase), and the echo train would monotonically approach zero with increasing TE, without a transient minimum or maximum.

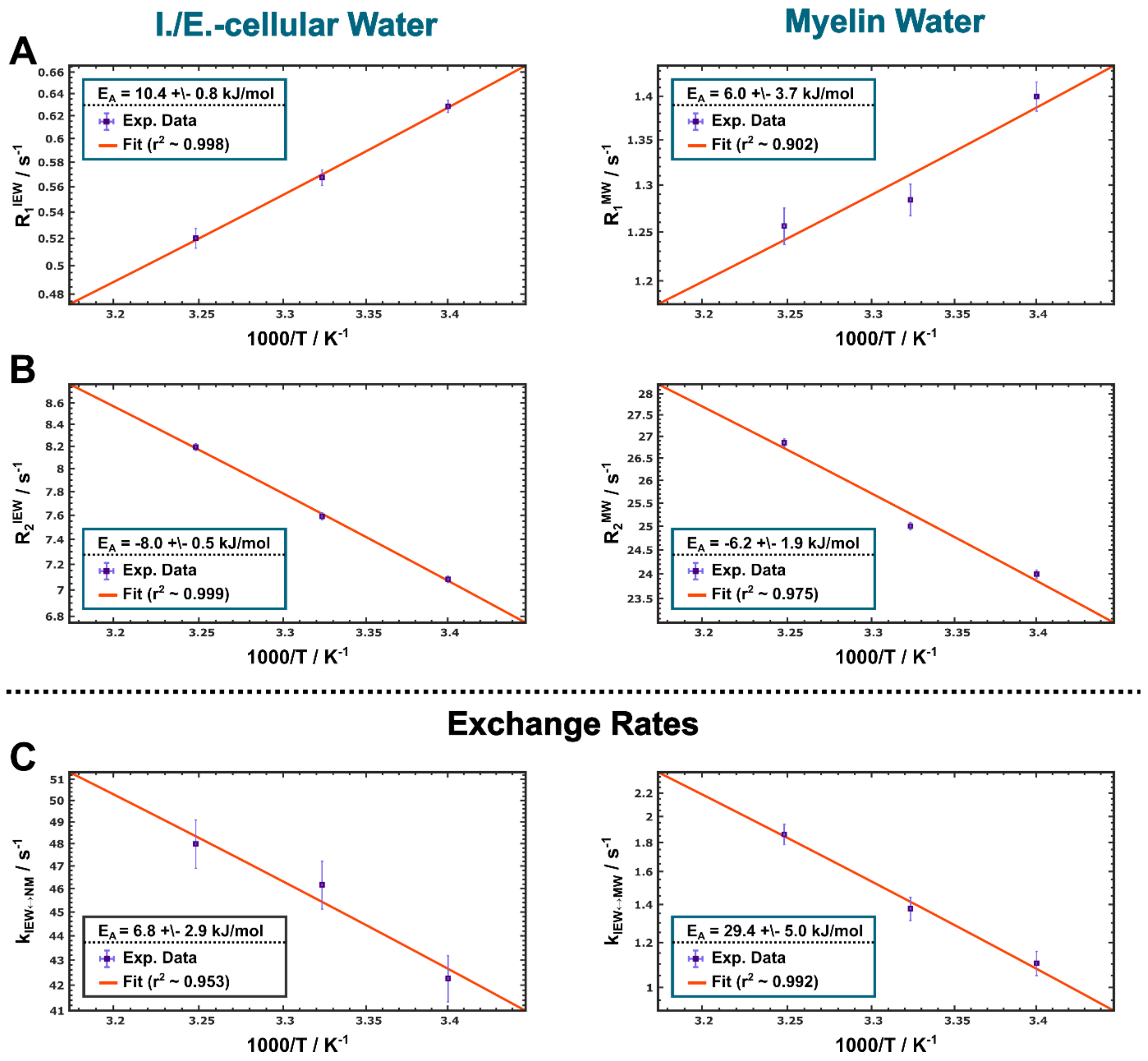


**Supplementary Figure S6. Results from Arrhenius analyses.** Shown are plots of the (logarithmic) longitudinal relaxation rates $R_1^{\mathrm{IEW}}$ and $R_1^{\mathrm{MW}}$ **(A)**, transverse relaxation rates $R_2^{\mathrm{IEW}}$ and $R_2^{\mathrm{MW}}$ **(B)**, and exchange rates $k_{\mathrm{IEW}\leftrightarrow\mathrm{NM}}$ and $k_{\mathrm{IEW}\leftrightarrow\mathrm{MW}}$ **(C)**. Blue symbols indicate the fitted parameters with confidence intervals (Table 2), red solid lines show fits to Eq. 14. The error bars of the experimental data (purple) indicate the confidence intervals as listed in Table 2. Uncertainty estimates for the fitted activating energies represent the 68% confidence interval.

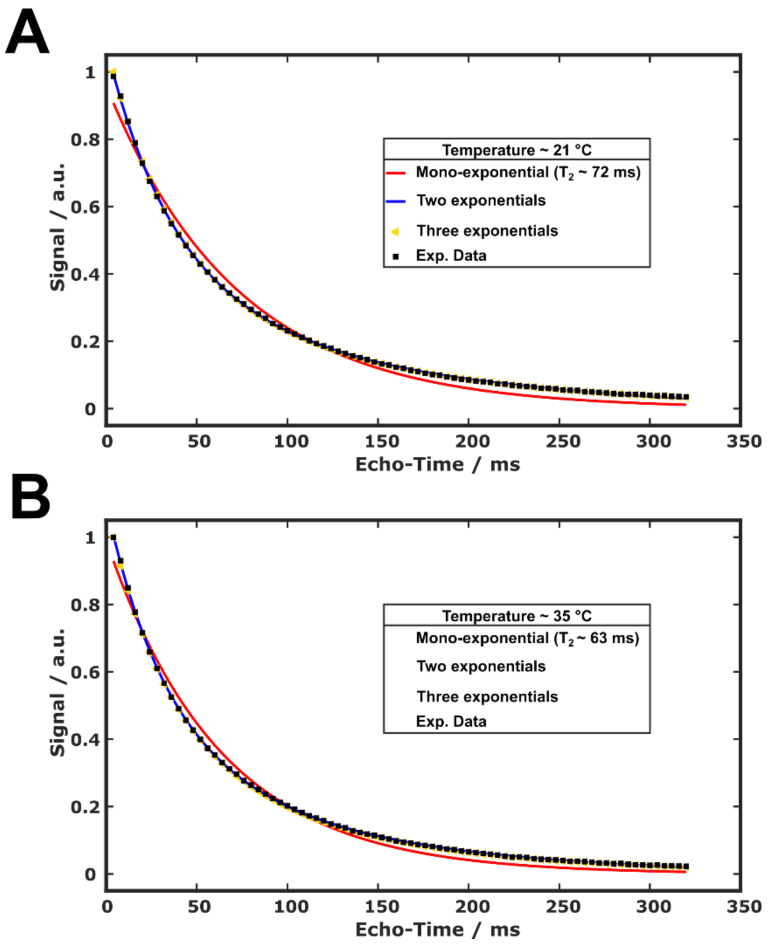


**Supplementary Figure S7. Exemplary multiexponential analysis of CPMG echo decays. (A)** Measurement at 21 °C; **(B)** measurement at 35 °C. The experimental data (black symbols) are well not described by a mono-exponential decay (red line). Excellent fits are obtained with two exponentials (blue line). Fits to three exponentials (yellow line) do not lead to further improvement.

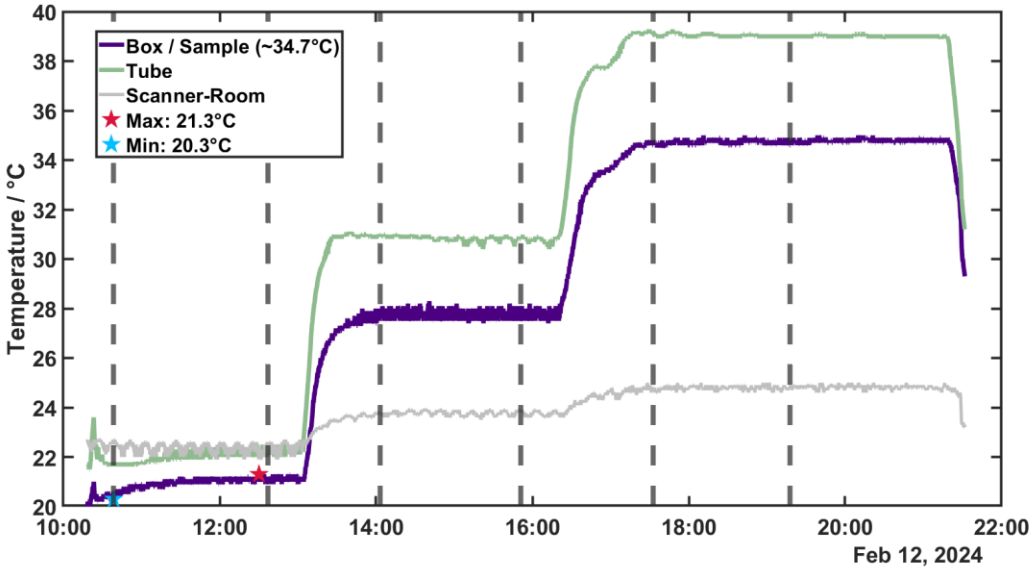


**Supplementary Figure S8. Temperature evolution during the experiment.** To monitor the temperature, fiberoptic sensors were integrated at three positions: *(i)* inside the wooden box enclosing the coil near the sample (purple), *(ii)* inside the tubing through which heated air was directed into the box (green), *(iii)* and inside the magnet room (gray). Dashed vertical gray lines indicate the start and end of the measurements for each temperature. Average temperatures during scanning inside the box were 21 °C, 28 °C, and 35 °C, which approximately correspond to the sample temperatures.
